# Identification and Immune Assessment of T Cell Epitopes in Five *Plasmodium falciparum* Blood Stage Antigens to Facilitate Vaccine Candidate Selection and Optimization

**DOI:** 10.3389/fimmu.2021.690348

**Published:** 2021-07-07

**Authors:** Vinayaka Kotraiah, Timothy W. Phares, Frances E. Terry, Pooja Hindocha, Sarah E. Silk, Carolyn M. Nielsen, Leonard Moise, Kenneth D. Tucker, Rebecca Ashfield, William D. Martin, Anne S. De Groot, Simon J. Draper, Gabriel M. Gutierrez, Amy R. Noe

**Affiliations:** ^1^ Leidos Life Sciences, Leidos Inc., Frederick, MD, United States; ^2^ EpiVax Inc., Providence, RI, United States; ^3^ The Jenner Institute, University of Oxford, Oxford, United Kingdom; ^4^ Center for Vaccines and Immunology, University of Georgia, Athens, GA, United States

**Keywords:** malaria, VAC063, class II T cell epitope, RH5, RIPR, CyRPA, EBA175, AMA1

## Abstract

The hurdles to effective blood stage malaria vaccine design include immune evasion tactics used by the parasite such as redundant invasion pathways and antigen variation among circulating parasite strains. While blood stage malaria vaccine development primarily focuses on eliciting optimal humoral responses capable of blocking erythrocyte invasion, clinically-tested *Plasmodium falciparum* (Pf) vaccines have not elicited sterile protection, in part due to the dramatically high levels of antibody needed. Recent development efforts with non-redundant, conserved blood stage antigens suggest both high antibody titer and rapid antibody binding kinetics are important efficacy factors. Based on the central role of helper CD4 T cells in development of strong, protective immune responses, we systematically analyzed the class II epitope content in five leading Pf blood stage antigens (RH5, CyRPA, RIPR, AMA1 and EBA175) using *in silico*, *in vitro*, and *ex vivo* methodologies. We employed *in silico* T cell epitope analysis to enable identification of 67 HLA-restricted class II epitope clusters predicted to bind a panel of nine HLA-DRB1 alleles. We assessed a subset of these for HLA-DRB1 allele binding *in vitro*, to verify the *in silico* predictions. All clusters assessed (40 clusters represented by 46 peptides) bound at least two HLA-DR alleles *in vitro*. The overall epitope prediction to *in vitro* HLA-DRB1 allele binding accuracy was 71%. Utilizing the set of RH5 class II epitope clusters (10 clusters represented by 12 peptides), we assessed stimulation of T cells collected from HLA-matched RH5 vaccinees using an IFN-γ T cell recall assay. All clusters demonstrated positive recall responses, with the highest responses – by percentage of responders and response magnitude – associated with clusters located in the N-terminal region of RH5. Finally, a statistically significant correlation between *in silico* epitope predictions and *ex vivo* IFN-γ recall response was found when accounting for HLA-DR matches between the epitope predictions and donor HLA phenotypes. This is the first comprehensive analysis of class II epitope content in RH5, CyRPA, RIPR, AMA1 and EBA175 accompanied by *in vitro* HLA binding validation for all five proteins and *ex vivo* T cell response confirmation for RH5.

## Introduction

Vaccine development against the blood stage of *Plasmodium falciparum* (Pf) continues to be of high interest, as this parasite stage is responsible for development of clinical illness in humans. Ideally, a successful blood stage vaccine should reduce morbidity and mortality, as well as limit transmission by interrupting development of gametocytes. Subunit vaccines targeting the blood stage are focused on a handful of merozoite antigens that have yet to advance into late-stage testing due to less than desired clinical efficacy, attributed to deficiencies in the elicited humoral response (1–3). While the blood stage is thought to be most vulnerable to inhibition by antibodies, malaria parasites have developed exceedingly effective mechanisms to circumvent otherwise lethal host antibody responses through the use of redundant invasion pathways, antigen complexity, and polymorphic antigens (4). High titers and fast kinetics of vaccine-induced blood stage parasite neutralizing antibodies against conserved non-redundant antigens have been identified as critical components of protective humoral responses (5–7).

The role of T cell immune responses against blood stage malaria infection has been elucidated through studies in mouse models and in clinical trials. Helper CD4 T cell responses are important for B cell stimulation and B cell-mediated clearance of parasites during models of chronic infection (8–10). Moreover, these cells can play a direct role in enabling the killing of activated phagocytes that have phagocytosed infected erythrocytes (11). Antigen-specific T cell responses have also been observed against viral-vectored malaria blood stage antigens in humans (12–17). Humoral responses induced by viral vector vaccines expressing malaria-specific and non-malaria antigens accompany induction of antigen-specific follicular T helper (Tfh) cells (12, 18, 19). For example, Tfh cells enhance memory and plasma B cell generation following influenza vaccination (20, 21). Further, frequencies of antigen-specific Tfh cells following vaccination with a malaria blood stage antigen RH5 correlate with peak anti-specific IgG concentration, frequency of antigen-specific memory B cells, and purified IgG *in vitro* neutralization activity (12). These findings strongly indicate that evaluation of T cell epitope content in vaccine targets is a critical step in vaccine design and optimization.

Malaria blood stage antigens present several vaccine design challenges due to the antigenic diversity that is the result of genetic variation and single nucleotide polymorphisms (SNPs) found among strains, and the propensity of these proteins to be inappropriately glycosylated in the expression systems used to generate vaccines. These challenges can be addressed during vaccine development as part of design and production optimization *via* some form of antigen engineering. To date, a systematic survey of the T cell epitope content in Pf blood stage antigens has not been conducted, even though multiple vaccines based on such targets (e.g., AMA1, EBA175 and RH5) have undergone clinical testing (14–17, 22–24). While the Immune Epitope Database and Analysis Resource (IEDB) includes a limited set of class I and II epitopes for AMA1 and EBA175, no T cell epitopes for the other three leading blood stage antigens RH5, CyRPA and RIPR have been curated from the scientific literature (25). *In silico* tools have been used to predict T cell epitopes in RH5 and EBA175 RII domain (26); however, the predicted epitopes were not validated for binding to human leukocyte antigen (HLA) molecules or in T cell activation assays. Therefore, a catalog of verified T cell epitopes would be invaluable to making an informed decision on optimizing antigen sequences for inclusion in a Pf blood stage vaccine.

In the current study we chose five key Pf blood stage antigens, EBA175, AMA1, CyRPA, RIPR, and RH5, and conducted *in silico* T cell epitope analysis utilizing tools from the iVAX toolkit (27). We then determined the *in vitro* binding affinities for the predicted epitopes, synthesized as peptides, to a set of HLA-DR alleles covering 95% of the worldwide population (28, 29). Finally, we assessed T cell recall responses with a subset of predicted HLA-DR-restricted epitopes from RH5. To our knowledge this is the first reported catalog of predicted epitopes in these major vaccine targets associated with *in vitro* HLA binding verification, as well as the first assessment of *ex vivo* T cell recall responses in Pf blood stage vaccinees utilizing peptides based on T cell epitope prediction.

## Materials and Methods

### *In Silico* T Cell Epitope Prediction and Analyses

The following accession numbers correspond to the Pf proteins used for class II epitope predictions: CZT98996-1 (AMA1), CAD49272-1 (CyRPA), CAD51055-2 (EBA175), CAD49275-1 (RH5) and CAB39049-1 (RIPR). The *in silico* analysis was conducted by EpiVax utilizing several tools from their iVAX toolkit (27, 30). Using the EpiMatrix tool, input amino acid sequences were parsed into overlapping 9-mer frames and each frame evaluated for predicted binding to a panel of nine class II HLA-DRB1 alleles (*0101, *0301, *0401, *0701, *0801, *0901, *1101, *1301, and *1501). EpiVax utilizes these alleles for their binding prediction algorithms as they represent functional allele supertypes (i.e., HLA alleles clustered into families based on the ability to bind peptides with related amino acid sequences), which facilitate evaluation of predictive immunity to over 95% of the global human population regarding HLA supertypes (28, 29). EpiVax normalizes these HLA binding predictions as EpiMatrix Z-scores (the output of this tool) to enable comparisons across alleles, and identifies significant frame “hits” by applying a Z-score cutoff of 1.64 (the top 5% of binding frames from a dataset of 10,000-random sequences) to signify a high probability of HLA allele binding. EpiVax also designates Z-scores in the top 1% of binding frames (applying a Z-score cutoff of 2.33) as hits with the highest probability of binding. The ClustiMer tool utilizes the EpiMatrix output to identify regions of high epitope density in the input sequences and define class II HLA epitope clusters, which consist of a binding core (containing a high density of predicted epitopes across the set of HLA-DR alleles evaluated) and flanking amino acids that facilitate HLA-DRB1 binding (31). The EpiMatrix outputs (predicted epitope sequences) were also evaluated for homology to the human genome (i.e., extent of “human-ness”) as an indicator of the potential to generate regulatory T cell (Treg) and/or other immunosuppressive responses. This homology analysis is performed using the JanusMatrix tool, which examines human sequence similarity with respect to the HLA and T cell receptor (TCR) faces of an epitope to flag sequences that could potentially elicit undesired autoimmune or Treg responses due to homology with sequences encoded by the human genome (32). The JanusMatrix (human homology) score of a given amino acid sequence indicates the number of potential immunosuppressive response triggers or flags, with higher JanusMatrix scores indicating a bias towards immune tolerance (33). Note that additional information concerning the role of the Treg repertoire in maintenance of self-tolerance can be found in Feng et al. (34). EpiVax provided detailed EpiMatrix outputs listing Z-scores for each frame across the complete set of HLA-DR alleles evaluated. We summarized these data as the highest Z-score and total number of predicted epitopes (EpiMatrix hit count) by HLA allele and Pf blood stage protein class II cluster.

### Peptide Synthesis

Predicted class II HLA epitope clusters were synthesized as peptides using solid phase chemistry, 9-fluoronylmethoxycarbonyl synthesis, by 21st Century Biochemicals. Peptide design included N-terminal acetyl group and C-terminal amino group caps and purity requirements specified >85% peptide purity as ascertained by HPLC, mass spectrometry and UV scan (ensuring purity, mass, and spectrum, respectively). Eleven peptides were synthesized without an N-terminal acetyl group to facilitate synthesis and purification. In order to establish a net charge, lysine flanking residues were also added to several peptides. In all cases, the amino acid content of each peptide was determined to enable reconstitution at highly accurate molarity.

### *In Vitro* HLA Binding Assays

The binding assay conducted by EpiVax yields an indirect measure of peptide-MHC affinity as it utilizes a competition assay format per the methodology described in Steere et al. (35). Briefly, a fluorescently-labeled, high-binding affinity, reference peptide (reconstituted in DMSO) and titrating concentrations of test peptide (reconstituted in DMSO) are incubated in a 96-well plate format with limiting concentrations of class II HLA monomers in aqueous buffer for 24-hours. Post incubation, the mixtures are moved to a 96-well plate coated with anti-HLA-DR antibody to capture HLA-peptide complexes. Time-resolved fluorescence measurement of the bound labeled reference peptide complex present in each mixture is detected with a europium-linked probe *via* fluorescent spectrophotometry using a SpectraMax M5 system. HLA binding affinity of each test peptide is expressed as the percent inhibition of reference peptide binding. Percent inhibition values (across the test peptide titration range) are used to calculate the half maximal inhibitory concentration (IC_50_) of each test peptide. For these studies, test peptides were assessed using a range of final concentrations from 100,000 nM to 100 nM. The panel of commercially-available HLA-DRB1 allele monomers used includes: *0101, *0301, *0401, *0701, *0801, *1101, *1301 and *1501. EpiVax established HLA-DRB1 binding affinity cutoffs were used to rank the IC_50_ values. In this assay, the experimental peptides were considered to bind with very high affinity if they inhibited 50% of control peptide binding at a concentration of 100 nM or less, high affinity if they inhibited 50% of control peptide binding at a concentration between 100 nM and 1,000 nM, and moderate affinity if they inhibited 50% of control peptide binding at a concentration between 1,000 nM and 10,000 nM. Low affinity peptides inhibited 50% of control peptide binding at concentrations between 10,000 nM and 100,000 nM. EpiVax has designated these rankings based on their experience with peptides in the very high to low affinity range as demonstrating positive measures of immunogenicity in follow-on *ex vivo* or *in vivo* experiments. Peptides that failed to inhibit at least 50% of control peptide binding at any concentration below 100,000 nM or that did not show a dose-dependent response pattern were considered to have negligible affinity or be non-binders.

### *Ex Vivo* T Cell Interferon (IFN)-γ Recall Assessments

As part of the VAC063 (36) and VAC057 clinical trials (14), blood samples were collected into lithium heparin-treated vacutainer blood collection systems (Becton Dickinson, UK) and peripheral blood mononuclear cells (PBMCs) isolated as previously described (15), frozen in fetal calf serum containing 10% DMSO, and stored in liquid nitrogen (vapor phase). For *ex vivo* assessments, peptides were reconstituted in 100% DMSO and used at 20µg/mL. ELISpot assays were performed as described in Payne et al. (14). Spots were counted using an ELISPOT counter (Autoimmun Diagnostika, Germany) and expressed as IFN-γ spot-forming counts (SFC) per million PBMCs. Background responses in unstimulated control wells were generally less than 20 SFC and were subtracted from test wells prior to data analysis. Notably, the VAC057 (ClinicalTrials.gov Identifier: NCT02181088) and VAC063 (ClinicalTrials.gov Identifier: NCT02927145) clinical trials were approved by the Oxford Research Ethics Committee A in the UK (REC references 14/SC/0120 and 16/SC/0345, respectively) as well as by the UK Medicines and Healthcare products Regulatory Agency (MHRA; references 21584/0331/001-0001 and 21584/0362/001-0001, respectively). All volunteers gave written informed consent.

### *In Silico* Prediction Accuracy and Correlation Analyses

Assessments of accuracy for *in silico* epitope prediction of *in vitro* HLA-DRB1 allele binding were calculated as the number of correct assessments divided by the number of all assessments where TN = true negatives, TP = true positives, FN = false negatives, and FP = false positives as per the following formula:

Accuracy=(TN+TP)/(TN+TP+FN+FP)

Assessments of correlation between *in silico* epitope predictions and *ex vivo* IFN-γ recall responses were calculated using the EpiVax individualized T cell epitope measure (iTEM) and Janus adjusted-iTEM (J-iTEM) tools (27, 37). The iTEM score considers the number of binding frames predicted for donor-specific HLA alleles to estimate a donor’s ability to respond to a particular peptide. The J-iTEM score applies deductions to iTEM scores based on cross-conservation with the human proteome according to JanusMatrix predictions; therefore, it reflects both epitope content and human cross-conservation.

## Results

### Experimental Workflow for *In Silico* Prediction and Laboratory Validation of Class II Epitopes

**Figure 1:**
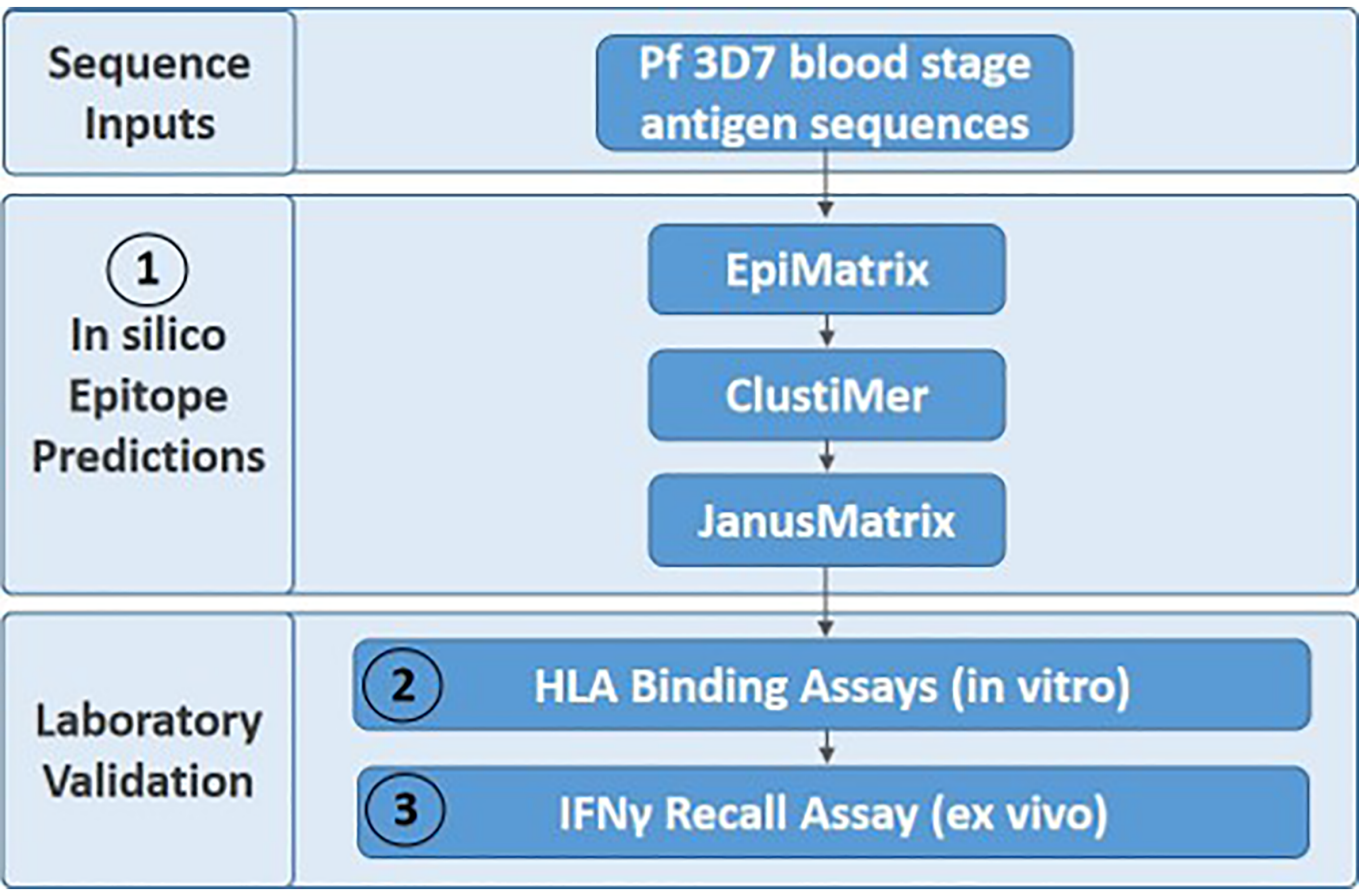
The three-step experimental workflow includes *in silico* (1), *in vitro* (2), and *ex vivo* (3) assessments.

We utilized Pf 3D7 sequences for the five blood stage antigens (**Table 1**) as inputs for the experimental workflow (**Figure 1**), starting with *in silico* analyses and moving to *in vitro* and *ex vivo* laboratory assessments in a step-wise manner as follows: (1) *in silico* analysis of the EBA175, AMA1, RIPR, CyRPA, and RH5 protein sequences was performed using the EpiMatrix tool to identify predicted class II T cell epitopes and these outputs were used in a ClustiMer analysis to define clusters of class II epitopes by identifying protein regions with high epitope density as well as in the JanusMatrix analysis to identify regions of human-ness within the predicted T cell epitope set and flag the potential for suppressive immune responses; (2) putative class II epitope clusters from the five proteins were synthesized as peptides and assessed *in vitro* for binding to HLA-DR alleles; and (3) the peptides from RH5 were then assessed for the ability to elicit T cell recall responses, *ex vivo*, using human PBMCs collected from RH5 clinical trial vaccinees.

**Table 1:** *In silico* analysis immunogenicity metrics show the highest scores for RH5, CyRPA, and RIPR as compared to EBA175 and AMA1.

Protein Name	Genbank ID	Protein Length (aa)	EPX Protein Score	JMX Protein Score
EBA175	CAD51055-2	1502	-32.74	0.87
AMA1	CZT98996-1	622	-32.10	0.82
RIPR	CAB39049-1	1086	-9.92	0.89
CyRPA	CAD49272-1	362	29.62	1.79
RH5	CAD49275-1	526	55.18	1.09

EpiMatrix (EPX) protein score represents the predicted class II epitope density for the protein overall, with scores ≥ 20 indicative of proteins with better than average immune potential relative epitope density. JanusMatrix (JMX) protein score represents an average of the number of epitopes flagged for the potential to elicit undesired autoimmune or Treg responses due to homology with the human proteome. While JanusMatrix cluster scores of ≥ 2.00 indicate epitope clusters with a higher than average overall potential for generating undesired autoimmune or Treg responses, the JanusMatrix protein score may underrepresent the presence of sequence cross-conservation with the human proteome, as this value is based on the average of the JanusMatrix cluster scores for each protein.

### *In Silico* T Cell Epitope Analysis of Five Pf Blood Stage Proteins

**Figure 2:**
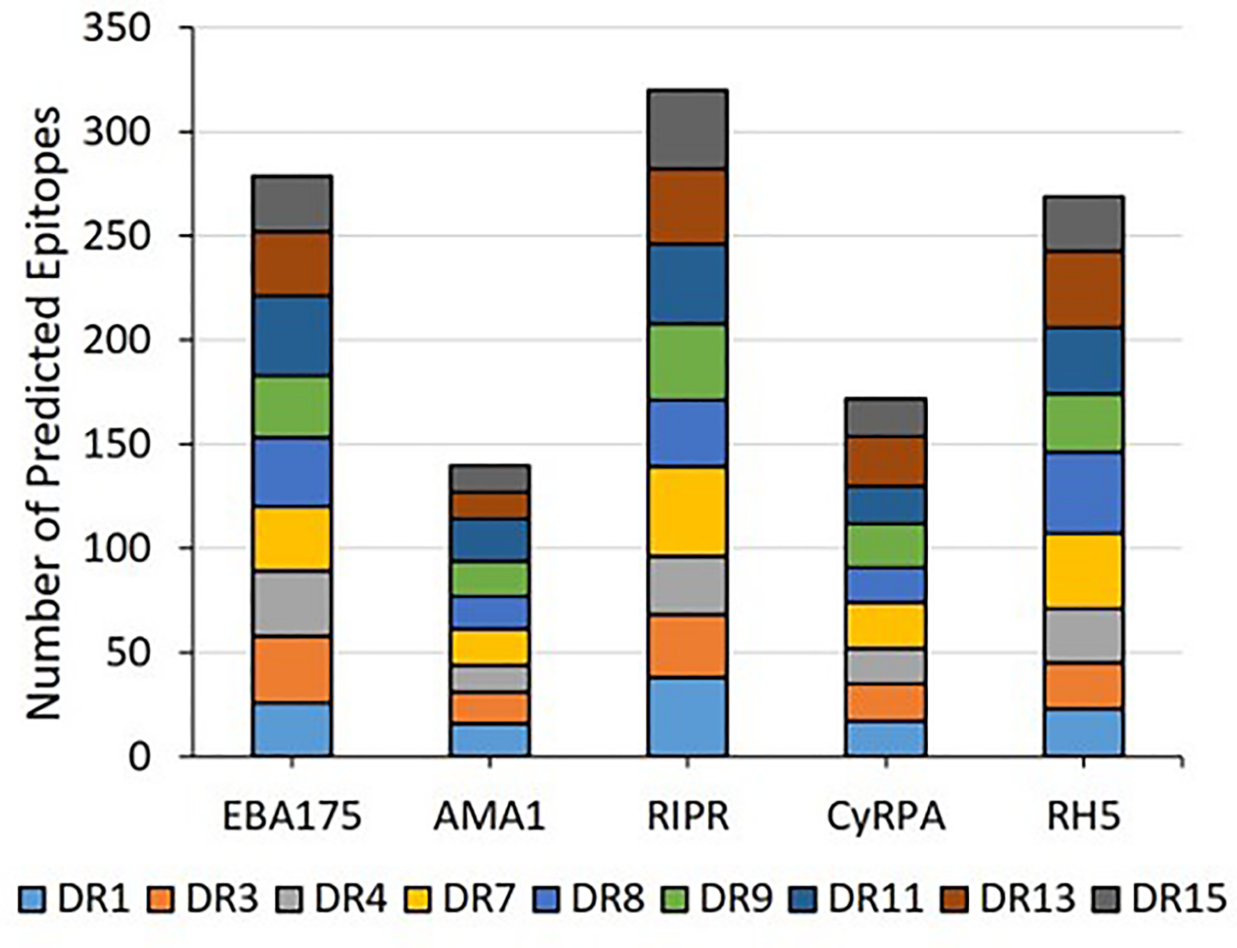
The predicted class II epitopes for the five Pf blood stage proteins are representative of all nine HLA-DRB1 alleles in the panel.

*In silico* analysis (step 1 of the workflow shown in **Figure 1**) of the five proteins was conducted to predict promiscuous class II T cell epitope clusters capable of binding multiple HLA-DRB1 alleles (DR1, DR3, DR4, DR8, DR9, DR11, DR13, and DR15). These specific alleles were selected because they are the most common HLA molecules within each of the HLA-DRB1 supertypes (38, 39) and are representative of >95% of human populations worldwide without the need to test each individual haplotype (28, 29). Output from the EpiMatrix and ClustiMer analyses included the overall density of predicted class II epitopes within each protein, represented as EpiMatrix protein score (**Table 1**), and the immunogenic potential for each identified cluster based on the number of predicted 9-mer HLA-DR binding frames (i.e., EpiMatrix hits) within each cluster, represented as EpiMatrix cluster score (cluster metrics are discussed in the below sections). Output from the JanusMatrix analysis included two metrics indicating the presence of flags for the potential to elicit undesired autoimmune or Treg responses within the set of predicted class II epitopes for each protein, based on sequence overlap with the human proteome (i.e., human-ness). These metrics are JanusMatrix cluster score, a measure of the number of flags in each identified cluster, and JanusMatrix protein score, an average of the JanusMatrix cluster scores for each protein (**Table 1**). In comparing the EpiMatrix results across the five proteins, we found the rank order from the highest to the lowest number of predicted epitopes by protein was RIPR > EBA175 > RH5 > CyRPA > AMA1, with all of the HLA-DRB1 alleles well-represented for each protein (**Figure 2**). Notably, as EpiMatrix protein score reflects epitope density across the full protein sequence, the larger proteins (EBA175, AMA1, and RIPR) ranked relatively low for this metric, while the smaller proteins (CyRPA and RH5) ranked relatively high (**Table 1**). An EpiMatrix protein score of ≥20 is considered good immune potential, which was reported for CyRPA and RH5. In terms of JanusMatrix protein scores, all five proteins demonstrate an overall lower than average number of human-ness flags, falling below the established cut-off score of 2.0 (40). However, as the JanusMatrix protein score is an average of the JanusMatrix cluster scores, individual clusters within a protein may still have scores above this threshold.

**Figure 3:**
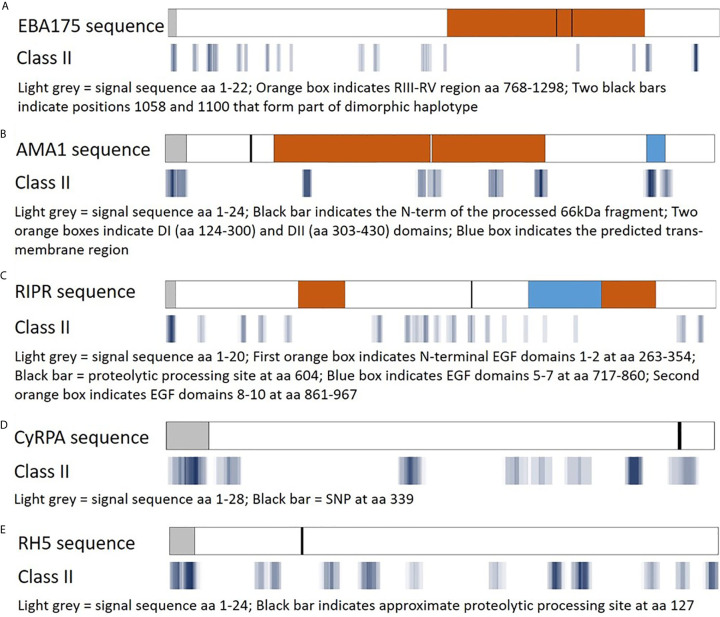
Predicted class II epitope coverage for Pf blood stage proteins. Major regions and features of EBA175 **(A)**, AMA1 **(B)**, RIPR **(C)**, CyRPA **(D)**, and RH5 **(E)** are shown in the top bar for each panel and aligned with predicted class II epitope clusters in the bottom bar for each panel. The predicted epitope binding frames are shown in blue with darker hues denoting a higher number of EpiMatrix hits (9-mer sequences with an EpiMatrix Z-score >1.64), lighter hues denoting fewer hits, and white spaces denoting no hits.

The EpiMatrix algorithm output is provided as Z-scores, indicating the predicted binding of each overlapping 9-mer in the amino acid sequence for each of the HLA-DRB1 alleles in the panel. The Z-scores equal to or greater than 1.64 represent the top 5% of predicted binding frames and signify a 9-mer with a high probability of HLA allele binding. As epitope clusters can contain more than one EpiMatrix hit for each allele, we summarize the EpiMatrix output for each cluster by providing the highest Z-score and total number of EpiMatrix hits per HLA-DRB1 allele. These data are provided for all five proteins in **Tables S1–S5**. Further, a composite representation of the predicted class II epitopes in each of the proteins (i.e., 9-mers with EpiMatrix Z-scores ≥ 1.64) representative of all nine HLA-DRB1 alleles is shown in **Figure 3**. This visualization of epitope density permits alignment of the predicted class II epitope clusters with major protein domains and/or features. For EBA175, the RIII-RV domain, which is well conserved relative to rest of the antigen, is remarkably poor in predicted class II epitopes (**Figure 3A**). For AMA1, the ectodomain contains the majority of epitope clusters with the DI and DII domains accounting for nearly all of the clusters outside the signal sequence and transmembrane region (**Figure 3B**). While the C-terminal EGF domains of RIPR have been identified as targets of neutralizing antibodies (41), these domains are relatively poor in class II epitope content. Rather, the majority of class II clusters are contained in the N-terminal fragment that is generated upon RIPR processing at the indicated site (**Figure 3C**). In CyRPA, the SNP at position 339 overlaps with a predicted class II cluster (**Figure 3D**). In RH5, the C-terminal proteolytic fragment contains most of the predicted class II clusters (**Figure 3E**). Notably, all five antigens have predicted class II epitope clusters in their signal sequences. As an increased number of human-ness flags can occur for this functional protein region, JanusMatrix cluster scores are relevant to inclusion of signal sequence content in vaccine candidate antigens. The JanusMatrix cluster score data for all five proteins are provided in **Tables S6–S10**.

### Selection of Class II Epitope Clusters for *In Vitro* Binding Assessments

Prior to conducting *in vitro* HLA binding assessments, a subset of the predicted class II clusters was synthesized as peptides. Downselection for peptide synthesis was performed in consideration of *in silico* analysis results, peptide synthesis feasibility flags, and peptide solubility metrics. Both EpiMatrix and JanusMatrix data were evaluated for each cluster of predicted epitopes (**Tables S1–S5**, **S6–S10**, respectively). Based on these metrics, several clusters were not considered for synthesis (e.g., RIPR cluster 594-614 due to a high JanusMatrix cluster score: 7.31, CyRPA cluster 151-172 due to a high JanusMatrix cluster score: 5.04, and RH5 cluster 224-246 due to a low EpiMatrix cluster score: 10.36). Additionally, a number of peptides were removed from consideration due to high hydrophobicity and/or anticipated problems related to peptide synthesis/purification, per guidance received from the peptide synthesis vendor. Peptide solubility was also considered such that the specific amino acid content of each peptide was determined to enable reconstitution at highly accurate molarity. In some cases, two peptides were synthesized for clusters with long lengths. In total, peptide synthesis was attempted for sequences in 12 of the 18 identified EBA175 clusters, 8 of the 9 identified AMA1 clusters, 18 of the 19 identified RIPR clusters, 6 of the 8 identified CyRPA clusters, and 10 of the 13 identified RH5 clusters; however, synthesis/purification failed for several of the AMA1, RIPR, and CyRPA peptides such that only 6, 10, and 2 clusters, respectively, were covered by the available peptides (**Table S11**). The sequences of all peptides are provided in **Table S12** (failed) and **Tables S13–S17** (synthesized) along with a designation indicating if the peptide sequence was trimmed from the ClustiMer output sequence and/or if charged amino acids were included to facilitate synthesis/purification. A total of 46 peptides covering 40 clusters were synthesized (**Table S11**) and assessed for HLA-DRB1 binding *in vitro*.

### *In Vitro* HLA Binding Analysis of Select Class II Epitope Clusters From Five Pf Blood Stage Proteins

**Figure 4:**
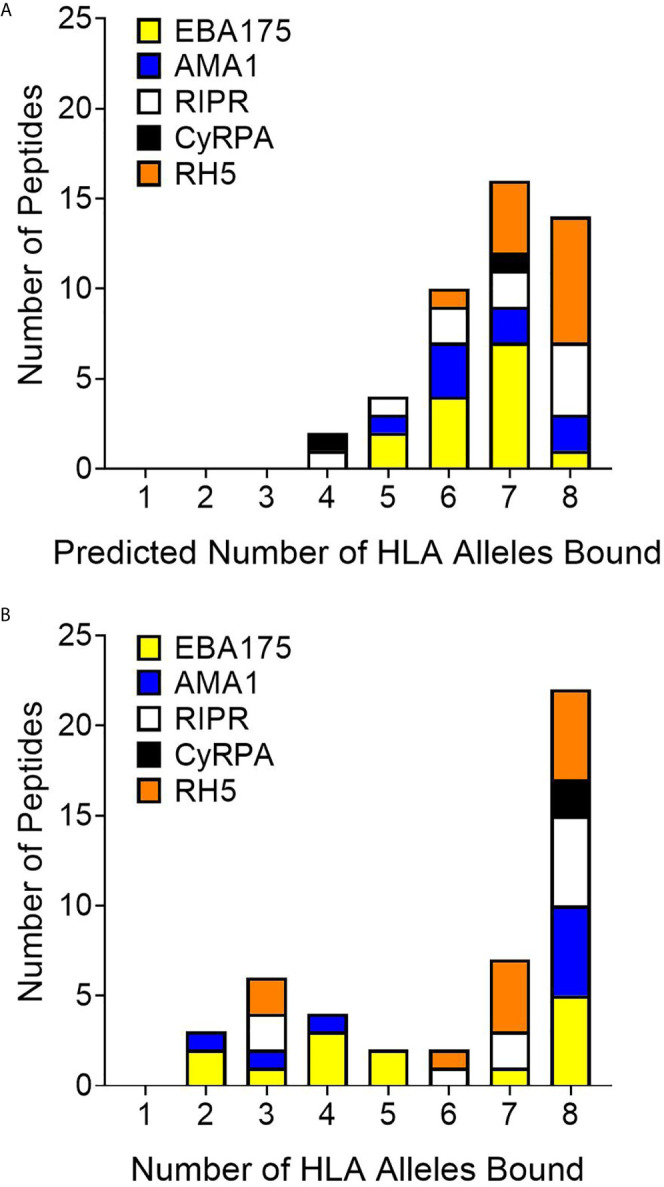
Comparison of predicted **(A)** to *in vitro* **(B)** HLA-DR allele binding for the 46 peptides representing 40 *in silico* predicted class II epitope clusters shows an increase from the prediction in the number of peptides that bind all eight HLA-DR alleles *in vitro*.

*In vitro* HLA binding assays (step 2 of workflow shown in **Figure 1**) were carried out with the synthesized peptides to verify the EpiMatrix outputs and provide a measure of accuracy for comparing *in silico*-predicted HLA-DRB1 allele binding to laboratory assessments of HLA-DRB1 allele binding. These assessments were conducted using a competition assay format whereby IC_50_ values were calculated based on the ability of the test peptide to compete with a labeled, high binding affinity reference peptide for HLA-DRB1 molecule binding. Sequences for the high affinity reference peptides and the negative control peptide, known not to bind any of the HLA-DRB1 molecules, are provided in **Table S18**. Notably, commercially-available HLA-DRB1 molecules were utilized in this assay and at the time of conduct, HLA-DRB1*0901 was not available; therefore, only eight alleles were included in the *in vitro* assessment panel. For all test peptides, the *in vitro* HLA binding analysis results (IC_50_ values) are provided in **Tables S13–S17**. Further, the associated IC_50_ value affinity rankings are identified in **Table S18**, with very high affinity to low affinity (i.e., IC_50_ values < 100,000nM) considered positive for HLA allele binding and IC_50_ values higher than this threshold considered negligible/negative for binding. Summary *in vitro* HLA binding assay results are provided such that the number of HLA alleles predicted (**Figure 4A**) is compared to the number of HLA alleles bound (**Figure 4B**) for the set of test peptides. When looking across the antigens, 22 of the 46 peptides tested were able to bind to all eight HLA-DR alleles while only 14 of the 46 peptides were predicted to bind all eight alleles. The number of peptides that bound all eight HLA alleles was more than that predicted for four of the proteins, EBA175, AMA1, RIPR, and CyRPA. Although the total number of RH5 peptides that bound eight HLA alleles was slightly reduced as compared to the prediction, this protein still had multiple peptides that bound all of the HLA alleles in the panel. The overall accuracy for *in silico* prediction of *in vitro* HLA binding was 71%, while protein specific accuracies were 61% for EBA175, 69% for AMA1, 79% for RIPR, 69% for CyRPA, and 79% for RH5 (**Table S19**).

### *Ex Vivo* IFN-γ Recall Assessments With RH5 Class II Clusters Utilizing RH5 Vaccinee Donor PBMCs

**Figure 5:**
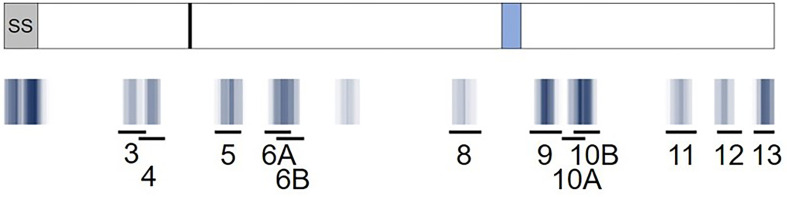
Predicted RH5 class II clusters (blue) aligned to the RH5 peptide set (black lines) assessed for *in vitro* HLA binding and *ex vivo* T cell IFN-γ recall responses. A visual representation of the epitope density of thirteen predicted class II epitope clusters for RH5 is provided and aligned to the RH5 peptide set (10 clusters represented by 12 peptides). The RH5 protein sequence and an overlay of key protein features are depicted in the top box. The signal sequence (SS, grey box), approximate proteolytic processing site at aa 127 (black line), and the loop separating the N- and C-terminal regions of the protein (light blue box) are shown. Darker blue hues denote clusters with higher epitope density, measured by number of EpiMatrix hits, while white areas denote no EpiMatrix hits. The specific locations of the twelve peptides tested are also shown. Note that the amino acid coordinates for the clusters corresponding to the peptides with the highest VAC063 donor responses, and that were also assessed with VAC057 donors, are as follows: peptide 3: 79-97; peptide 4: 92-110; and peptide 6A: 179-205.

**Figure 6:**
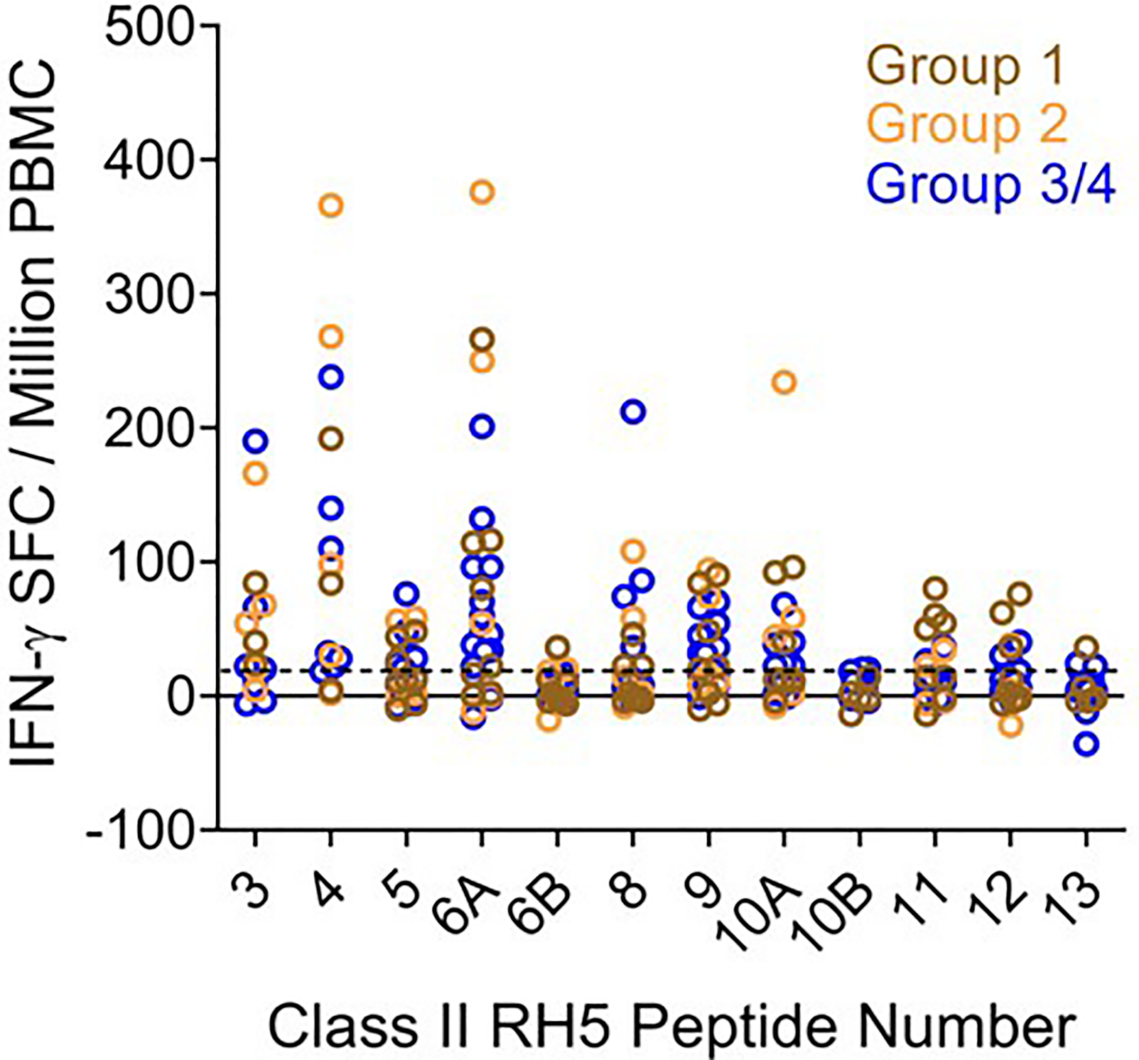
*Ex vivo* IFN-γ recall responses to predicted RH5 class II epitope clusters with HLA-matched VAC063 vaccinee donor PBMCs demonstrate the highest T cell responses in clusters found in the N-terminal region of RH5. Individual data points (colored circles) represent vaccinee samples from different dosing regimen groups (**Table S20**). Peptide numbers indicate the predicted class II cluster number (numbered sequentially from the N- to C-terminus). The established positivity cut-off (dotted line) was ≥ 20 SFC/million PMBCs.

To determine if the class II clusters identified through *in silico* analysis (and verified *via in vitro* binding assays) would elicit T cell recall immune responses, we utilized the set of RH5 peptides in *ex vivo* assessments of IFN-γ T cell recall responses (step 3 of workflow in **Figure 1**) with PBMCs collected from VAC063 clinical trial volunteers (36). All PBMC donors from the VAC063 trial were vaccinated with the recombinant protein-based RH5 vaccine candidate RH5.1 (42), formulated in AS01_B_ adjuvant. Further, all donors participated in the dose-escalation study arm (i.e., these volunteers received different amounts of antigen depending on group assignment and were not challenged) and *ex vivo* recall responses were assessed in samples collected 14 days after the second vaccine administration. Donors from four groups were included in the recall response assessment, with each volunteer having received two administrations of formulated RH5.1 (at study days 0 and 28) as follows: group 1 received 2 μg of RH5.1 per dose, group 2 received of 10 μg of RH5.1 per dose, and groups 3 and 4 received 50 μg of RH5.1 per dose (**Table S20**). For the data analysis, the background response (media only control) was subtracted from all test samples and a positivity cut-off of ≥20 SFC/million PBMCs was established to evaluate the background subtracted data. Although not all of the HLA alleles were well-represented across the VAC063 volunteers and PBMC sample limitations impacted the number of peptides that could be assessed across the available donors, positive recall responses were seen to all of the peptides and a high percentage of HLA-matched donors responded to several peptides (**Table 2**). In particular, a high percentage of donors responded to peptides representing the class II clusters located in the N-terminal region of RH5 as compared to those located in the C-terminal region of the protein (see **Figure 5** for alignment of the peptides and clusters relative to the RH5 protein sequence). Further, the magnitude of HLA-matched donor responses was also highest with the peptides representing the N-terminal clusters (**Figure 6**). Differential recall responses were not found based on donor group assignment; however, it should be noted that other measures of immunogenicity (i.e., ELISA) also did not differ among the vaccinee groups after two doses (36).

**Table 2:** High percentages of VAC063 donors, by matched HLA-DR allele, recall RH5 class II epitope clusters located in the N-terminal region of the protein.

Peptide #	HLA Allele								
	DR1	DR3	DR4	DR7	DR8	DR9	DR11	DR13	DR15
**3**	**100%**	**75%**	**75%**	**60%**	**ND**	**ND**	**0%**	**50%**	**50%**
**4**	**100%**	**75%**	**100%**	**100%**	**ND**	**ND**	**0%**	**50%**	**50%**
**5**	**80%**	**38%**	**14%**	**70%**	**100%**	**100%**	**0%**	**43%**	**50%**
**6A**	**100%**	**50%**	**71%**	**100%**	**100%**	**100%**	**33%**	**71%**	**50%**
**6B**	**60%**	**0%**	**14%**	**20%**	**100%**	**0%**	**0%**	**43%**	**13%**
**8**	**60%**	**25%**	**29%**	**30%**	**100%**	**0%**	**0%**	**43%**	**13%**
**9**	**80%**	**38%**	**43%**	**60%**	**100%**	**100%**	**33%**	**29%**	**63%**
**10A**	**60%**	**38%**	**57%**	**30%**	**100%**	**0%**	**33%**	**43%**	**50%**
**10B**	**0%**	**0%**	**0%**	**17%**	**0%**	**0%**	**0%**	**40%**	**20%**
**11**	**40%**	**38%**	**71%**	**30%**	**0%**	**0%**	**0%**	**14%**	**13%**
**12**	**40%**	**50%**	**14%**	**30%**	**0%**	**0%**	**0%**	**14%**	**13%**
**13**	**0%**	**20%**	**0%**	**0%**	**0%**	**0%**	**0%**	**60%**	**40%**
**N^a^**	**3**	**4**	**4**	**5**	**0**	**0**	**2**	**4**	**4**
**N^b^**	**5**	**8**	**7**	**10**	**1**	**1**	**3**	**7**	**8**
**N^c^**	**3**	**5**	**4**	**6**	**1**	**1**	**2**	**5**	**5**

Ex vivo IFN-γ recall responses to RH5 class II clusters were found for all clusters assessed (10 clusters represented by 12 peptides) with the highest percent of donor responses seen across the matched HLA-DR alleles for clusters located in the N-terminus of the protein (clusters 3-8, which are aligned to peptide number). The threshold positivity cut-off was ≥ 20 SFC/million PBMCs for media subtracted recall responses. The percentage of donors (RH5.1 vaccinees) responding, by HLA-DR allele for each peptide, is shown numerically along with color-coding to indicate higher percentages of responders with darker blues. The number of HLA-matched donors (N) for each allele is shown in the bottom rows. Due to limitations on available PBMCs, it was not possible to assess recall responses for all peptides across the full donor set. The number of HLA-matched donors was low for certain alleles (e.g., only one matched donor for HLA-DR8 and one matched donor for HLA-DR9). ND = not determined due to limitation of PBMCs.

N^a^ – number of HLA-matched donors assessed with peptides 3 & 4; 16 donors. N^b^ – number of HLA-matched donors assessed with peptides 5, 6A, 6B, 8, 9, 10A, 11, & 12; 28 donors. N^c^ – number of HLA-matched donors assessed with peptides 10B & 13; 16 donors.

To determine if similarly high recall responses might be found in RH5 vaccinees from the VAC057 clinical trial, peptides representing three of the N-terminal clusters (clusters 3, 4, and 6) were also assessed in HLA-matched donors from this study. Notably, RH5 was delivered *via* viral vector in VAC057 and differences in immunogenicity between recombinant and viral vectored RH5 have been shown (12). *Ex vivo* assessments of IFN-γ T cell recall responses were conducted using donor samples collected 14 days after the VAC057 vaccinees had received a single dose (5x10^10^ viral particles) of ChAd63 RH5 (**Table S21**). The *ex vivo* study results showed similarly good recall responses to peptides 3, 4, and 6A with cells from the VAC057 HLA-matched donors as those from VAC063 donors (**Table 3**). Further, similar recall response magnitudes were also seen with VAC057 HLA-matched donor cells (**Figure S1**).

**Table 3:** High percentages of VAC057 donors, by matched HLA-DR allele, recall three RH5 class II clusters located in the N-terminal region of the protein.

Peptide #	HLA Allele								
	DR1	DR3	DR4	DR7	DR8	DR9	DR11	DR13	DR15
**3**	**33%**	**25%**	**67%**	**40%**	**ND**	**ND**	**50%**	**100%**	**100%**
**4**	**100%**	**75%**	**100%**	**60%**	**ND**	**ND**	**50%**	**100%**	**50%**
**6A**	**67%**	**75%**	**83%**	**40%**	**ND**	**ND**	**50%**	**100%**	**25%**
**N**	**3**	**4**	**6**	**5**	**0**	**0**	**2**	**2**	**4**

High percentages of ex vivo IFN-γ recall responses were confirmed to three RH5 class II clusters (clusters 3, 4, and 6 represented by 3 peptides) in the N-terminal region of RH5 using HLA-matched donors from the VAC057 clinical trial (ChAd63 RH5 vaccinees). The threshold positivity cut-off was ≥ 20 SFC/million PBMCs for media subtracted recall responses. Percentage of donors responding, by HLA allele for each peptide, is shown numerically along with color-coding to indicate higher percentages of responders with darker blues. The number of HLA-matched donors (N) for each allele is shown in the bottom row (14 donors total). Note that the number of HLA-matched donors was low for certain alleles and no HLA-matched donor PBMCs were available for DR8 and DR9. ND = not determined due to no HLA-matched donor PBMCs available.

### Correlation of *In Silico* Epitope Predictions to *Ex Vivo* Recall Response Assessments

**Figure 7:**
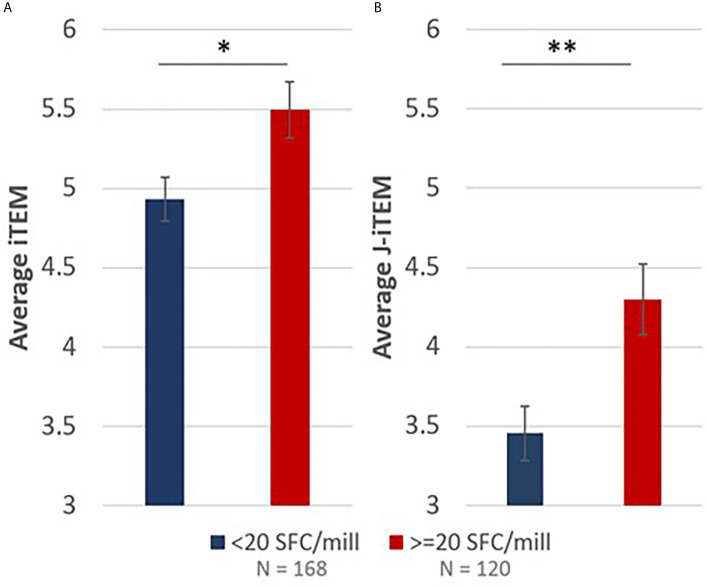
iTEM **(A)** and J-iTEM **(B)** calculations demonstrate a statistically significant positive correlation between *in silico* EpiMatrix and JanusMatrix predictions and *ex vivo* IFN-γ recall responses. Error bars represent standard error. * indicates p < 0.05 and ** indicates p < 0.01.

To understand if the RH5 *in silico* epitope predictions (EpiMatrix scores) correlated with the *ex vivo* T cell recall responses in RH5 vaccinees (based on the donor HLA haplotype), we calculated and compared iTEM scores for positive recall responses (≥ 20 SFC/million PBMCs) to that of negative recall responses (< 20 SFC/million PBMCs). The iTEM score aligns the presence or absence of predicted class II epitopes in a peptide to the HLA haplotype for each donor. Therefore, higher average iTEM scores are achieved when HLA-matched positive and negative donor recall responses align with *in silico* HLA binding/non-binding predictions for a specific peptide sequence (i.e., there is alignment between the true positives and true negatives for predictions and responses). In comparing average iTEM scores for positive recall responses to negative recall responses, a statistically-significant increase (p<0.05) in average iTEM score was found for the positive recall response set, indicating a positive correlation between *in silico* HLA-matched epitope predictions and positive *ex vivo* recall responses (**Figure 7A**). Moving one step further, we also calculated J-iTEM scores for the positive and negative recall responses. The J-iTEM score takes into account JanusMatrix scores for the peptides (e.g., a reduced recall response may result from the presence of Treg cells); therefore, J-iTEM scores reflect a reduction in iTEM scores for those peptides with a higher JanusMatrix cluster score. In comparing average J-iTEM scores for the positive recall responses to that for negative recall responses, a statistically-significant increase (p < 0.01) in average J-iTEM score was found for the positive recall response set, indicating a positive correlation between *in silico* HLA-matched epitope predictions, when accounting for possible reductions in overall response due to sequence overlap with the human proteome, and positive *ex vivo* recall responses (**Figure 7B**).

## Discussion

We carried out systematic identification and verification of predicted class II T cell epitopes for five Pf blood stage antigen vaccine targets to better enable blood stage malaria vaccine design through identification and validation of highly promiscuous HLA-DRB1-restricted epitope clusters. Our workflow included *in silico* epitope prediction and analysis using tools from the iVAX toolkit and laboratory verification using *in vitro* HLA-DRB1 binding assays and *ex vivo* T cell recall assays. In total, we identified 67 predicted class II epitope clusters across the five proteins – EBA175 (18), AMA1 (9), RIPR (19), CyRPA (8), and RH5 (13). In evaluating metrics for epitope immunogenicity and density relevant to the overall predicted class II epitope content for the proteins, CyRPA and RH5 had the top EpiMatrix protein scores, 29.62 and 55.18, respectively, with scores >20 considered above average for immunogenicity and a score of zero equal to random expectation. The EpiMatrix protein score for RIPR was -9.92, while both EBA175 and AMA1 had scores below −30. Notably, the EpiMatrix protein score for RH5 is higher than several well-known immunogens including Epstein-Barr virus-BKRF3, influenza H1N1 HA, and tetanus toxin, all of which have EpiMatrix protein scores in the 20 to 40 range (27). Further, the three components of the RCR complex, RH5, CyRPA and RIPR, had the highest EpiMatrix protein scores of the five proteins assessed, emphasizing their importance as blood stage vaccine candidates individually, in combination, or as a formulation of the pre-formed RCR complex (43).

In looking at the more granular level of epitope cluster metrics, the vast majority of the clusters identified through the EpiMatrix and ClustiMer tool outputs were predicted to bind seven or more of the nine HLA-DRB1 alleles in the analysis panel (*0101, *0301, *0401, *0701, *0801, *0901, *1101, *1301, and *1501). The exceptions to this were found in EBA175 and AMA1, with one cluster each predicted to bind only six of the HLA-DRB1 alleles, and in RIPR, with three clusters predicted to bind only six and another three clusters predicted to bind only five of the HLA-DRB1 alleles. We also evaluated the predicted epitopes for the presence of sequence cross-conservation with the human proteome, as this is considered a flag for potential Treg or tolerance responses. The JanusMatrix analysis scores the homology of TCR facing residues of the predicted class II epitopes to human protein sequences that are TCR facing residues when presented in the context of a MHC allele. A higher overall JanusMatrix score indicates greater chance that an epitope might be recognized as self and thus activate Treg cells and/or lead to down-regulation or toleration of vaccine-induced, antigen-specific immune responses (44–46). A total of eight predicted epitope clusters had JanusMatrix cluster scores above 2.0, which indicates a higher than average amount of human sequence overlap in the TCR facing residues. These flagged clusters were found on EBA175 (cluster 3: 101-122 and cluster 9: 328-344, JMX clusters scores 2.58 and 2.14, respectively), RIPR (cluster 12: 594-614, JMX cluster score 7.31), CyRPA (cluster 1: 2-30, cluster 3: 151-172, and cluster 6: 261-284, JMX clusters scores 3.67, 5.04, and 2.23, respectively), and RH5 (cluster 1: 1-15 and cluster 10: 382-4082, JMX cluster scores 2.40 and 3.29, respectively). For CyRPA and RH5, high JanusMatrix cluster scores were found for class II clusters that align with the signal sequence.

Using the EpiMatrix and JanusMatrix outputs as well as protein synthesis flags and peptide solubility metrics, we down selected cluster sequences for peptide synthesis. Of the 67 predicted class II clusters, peptide synthesis was attempted for 54 cluster sequences. However, due to synthesis failures, the total number of peptides synthesized was 46 (representing 40 clusters). These peptides were utilized for *in vitro* HLA-DRB1 binding assessments, the second step in our workflow, as a verification of the *in silico* predictions with all of the peptides assessed for binding using a panel of eight HLA-DRB1 molecules (*0101, *0301, *0401, *0701, *0801, *1101, *1301, and *1501). All of the 46 peptides bound at least two of the HLA-DRB1 alleles and 80% (37 of the 46) bound four or more of the HLA-DRB1 alleles. Further, while only 14 of the peptides were predicted to bind all eight of the alleles, 22 of the peptides bound all eight of the alleles *in vitro*. This is consistent with the observation that many HLA-DR alleles share peptide-binding preferences (47).

An estimation of accuracy of the EpiMatrix algorithm is useful to determine the confidence level that can be placed on predicted epitopes during antigen selection and optimization. Accordingly, taking into account the *in vitro* HLA binding results, the false positive and false negative rates for epitope prediction were calculated as a measure of prediction accuracy. In an *in vitro* system utilizing synthetic peptides and synthetic HLA molecules, some discrepancy between predicted and observed outcomes is to be expected. For this reason, the EpiMatrix Z-score hit cut-off of 1.64 is configured to select some peptides expected to bind at lower affinity and also cast a wide net to reduce the possibility of missing high-quality targets (type 2 error). It is interesting to note that the majority of the false negative counts are associated with EpiMatrix Z-scores between 1.28 and 1.64, which EpiVax considers “elevated” but not positive. Peptides scoring in this range sometimes bind HLA, but in general, less often and at lower affinity. Overall, we achieved 71% accuracy in predicting *in vitro* HLA binding with *in silico* epitope predictions.

The third step in our workflow was to conduct *ex vivo* T cell stimulation assays with the RH5 peptide set (12 peptides covering 10 predicted class II clusters), to determine if these could elicit IFN-γ recall responses from the CD4 fraction present in PBMCs collected from RH5 vaccinees from two clinical studies, VAC063 (recombinant RH5.1 formulated in AS01_B_ adjuvant) and VAC057 (viral-vectored RH5). For VAC063, samples from 28 HLA-matched donors were tested, while for VAC057, samples from 14 HLA-matched donors were tested. All 12 peptides generated positive responses at the ≥20 SFC/million PBMC positivity threshold (data were background-subtracted prior to analysis) with the number and magnitude of HLA-matched VAC063 donor responses higher for peptides representing class II clusters in the N-terminal region of RH5 and lower for peptides representing class II clusters in the C-terminal region of the protein. The highest responses – by percentage and magnitude of response – were seen in peptides representing clusters nearest to the N-terminus and these high recall responses were verified in HLA-match VAC057 donors. Of note is that two of the RH5 class II clusters we evaluated (clusters 3 and 4) are located in the processed 45kDa protein fragment and both demonstrated some of the highest HLA-matched donor IFN-γ recall responses seen in the *ex vivo* assessment. Further, while RH5 is very highly conserved across parasite isolates, four of five RH5 SNPs with allele frequencies >9% (43) are located within two of the clusters with the highest recall responses as follows: SNPs H148D and Y147H are located in cluster 3 and SNPs S197Y and C203Y are located in cluster 6. For the two peptides demonstrating the lowest responses, one (peptide 10B) performed well in the *in vitro* HLA binding assay (positive for seven of eight HLA alleles) but had a high JanusMatrix cluster score (3.29) and the other (peptide 13) performed poorly in the *in vitro* binding assay (positive for three of eight HLA alleles) and had a moderate but lower than average JanusMatrix cluster score (1.75). Regarding the former, the data suggest that the low recall responses to this peptide may be due to an immune suppressive T cell response; however, confirmation of this is needed through evaluation of appropriate markers (e.g., IL-10).

Assessments of correlation between the *in silico* predictions and *ex vivo* VAC063 IFN-γ T cell recall responses were determined using the iTEM and J-iTEM tools from the iVAX toolkit. We found a statistically significant increase in average iTEM score with positive donor recall responses as compared to negative donor recall responses when considering HLA-matches between the predicted HLA-restricted epitopes (in the peptides) compared to donor HLA phenotypes (p<0.05). Further, when we accounted for the possibility of lower recall responses due to the presence of suppressive T cells (i.e., conducted the J-iTEM analysis, which considers JanusMatrix scores), the statistical significance comparing positive to negative donor recall responses was even higher (p < 0 .01). These results substantiate our workflow and the utility of *in silico* analysis tools for T cell epitope predictions.

While we primarily focused on predictive analysis and assessment of class II epitope clusters, we cannot rule out the possibility that class I epitopes may play a role in robust immune responses to blood stage malaria infection as well as the possibility that elicitation of CD8 T cell responses to blood stage epitopes may be relevant based on the specific vaccine platform. Further, parasite-specific CD8 T cells have been shown to be important for mediating long-term immunity against the blood stage in a model of chronic malaria (48, 49) as well as in the recognition and killing of *P. vivax*-infected reticulocytes (50). Additionally, CD8 T cell responses may be relevant for blood stage antigens also expressed in the liver stage of the parasite, such as AMA1 (51). Therefore, the prediction and evaluation of class I HLA-A and HLA-B epitopes was included as part of our umbrella strategy for evaluation of malaria blood stage vaccine targets. *In silico* epitope predictions across a panel of six class I HLA-A and HLA-B alleles (A*0101, A*0201, A*0301, A*2402, B*0702, B*4402) were performed for the five blood stage antigens and included assessment of *in vitro* HLA binding to 212 of the predicted epitopes synthesized as peptides (**Tables S23–S27**). Overall, 75% of the predicted class I epitopes that were tested bound the class I HLA allele (*in vitro*) that they were predicted to bind (**Table S28**). The RH5 HLA-A*0201 peptide set (14 peptides) was assessed for CD8 T cell response stimulation in the *ex vivo* IFN-γ recall assay with eleven HLA-A*0201 matched donors, five from VAC063 and six from VAC057. Five of the peptides demonstrated positive responses at the ≥20 SFC/million PBMC positivity threshold (data were background-subtracted prior to analysis) (**Table S29**). Notably, all five of these epitopes were located toward the C-terminus of RH5. The highest magnitude responses were seen to peptide RH5_492, to which four VAC057 donors responded (**Figure S2**). This peptide overlaps the RH5 class II cluster 12.

To our knowledge, this is the first comprehensive *in silico* prediction of epitope content in RH5, CyRPA, RIPR, AMA1 and EBA175 accompanied by laboratory validation of the predicted epitopes through *in vitro* HLA binding and *ex vivo* T cell simulation assessments. The analyses presented herein demonstrate the utility of *in silico* epitope prediction to identify sets of epitopes for evaluation as part of vaccine design and optimization. Further, while we achieved relatively high accuracy in predicting *in vitro* HLA binding, we note that, the *ex vivo* recall response data generate the most actionable information for use in vaccine design as these provide an understanding of which T cell epitopes are likely to elicit T cell responses in target human populations. Our analysis workflow can thus provide actionable data for considering and preserving T cell epitope content during selection and optimization of antigens for blood stage malaria vaccine development.

## Data Availability Statement

The original contributions presented in the study are included in the article/**Supplementary Material**. Further inquiries can be directed to the corresponding author.

## Ethics Statement

The studies involving human participants were reviewed and approved by Oxford Research Ethics Committee A in the UK (REC references 14/SC/0120 and 16/SC/0345, respectively) as well as by the UK Medicines and Healthcare products Regulatory Agency (MHRA; references 21584/0331/001-0001 and 21584/0362/001-0001, respectively). The patients/participants provided their written informed consent to participate in this study.

## Author Contributions

Overall conceptualization and study designs were contributed by AN, VK, KT, TP and GG. *In silico* analyses, *in vitro* HLA binding assays, data and statistical analysis were performed by FT, LM, and PH, with supervision by WM and ADG. *Ex vivo* lymphocyte activation assays were performed and analyzed by SS and CN, with supervision by RA and SD. Project management, data organization and formal analyses were performed by VK and AN. Manuscript writing was performed by VK, AN, and TP. Manuscript editing and review were performed by FT, LM, ADG, CN and SD. All authors contributed to the article and approved the submitted version.

## Funding

These studies were made possible through support provided by the Office of Infectious Diseases, Bureau for Global Health, U.S. Agency for International Development (https://www.usaid.gov), under the terms of the Malaria Vaccine Development Program (MVDP) Contract AID-OAA-C-15-00071, for which Leidos, Inc. is the prime contractor. The funders approved study plans but had no direct role in development of study designs, data collection/analysis, or preparation of the manuscript. CN is a Wellcome Trust Sir Henry Wellcome Postdoctoral Fellow [209200/Z/17/Z]. SD is a Jenner Investigator and a Wellcome Trust Senior Fellow [106917/Z/15/Z].

## Disclaimer

The opinions expressed herein are those of the authors and do not necessarily reflect the views of the U.S. Agency for International Development.

## Conflict of Interest

AN, TP, KT, VK, and GG are employees of Leidos, Inc., the prime contractor for the USAID Malaria Vaccine Development Program (MVDP) Contract AID-OAA-C-15-00071. FT, PH, LM, WM, and ADG are employees of EpiVax, Inc., an MVDP subcontractor. PH is a previous employee of EpiVax, Inc. SD, SS, CN, and RA are employees of the University of Oxford, an MVDP subcontractor. SD is a named inventor on patent applications relating to the RH5-based vaccines used in the VAC057 and VAC063 clinical studies.
