# Supplementary material for: Identification and Immune Assessment of T Cell Epitopes in Five Plasmodium falciparum Blood Stage Antigens to Facilitate Vaccine Candidate Selection and Optimization
Source: Front Immunol. 2021 Jul 7;12:690348. doi: 10.3389/fimmu.2021.690348 (PMC8294059; doi:10.3389/fimmu.2021.690348)
Supplement: Supplementary file 1 [file DataSheet_1.docx]

Supplementary Material

# Overview

This supplement includes data and information regarding the class II epitope identification and analysis (**Section 3**) and the class I epitope identification and analysis (**Section 4**) for the *Plasmodium falciparum* (Pf) proteins EBA175, AMA1, RIPR, CyRPA, and RH5. With regard to the class I analysis, these data were not the primary focus of our efforts to better elucidate Pf blood stage vaccine design and optimization but rather were included in our overall strategy for informative purposes, as we cannot rule out the possibility that CD8 T cell response may have some role in protective immunity. We have provided the class I epitope data herein to make these available to the broader research community.

# Table of Contents

[1 Overview 1](#_Toc70848758)

[2 Table of Contents 1](#_Toc70848759)

[3 Class II Epitope Prediction and Analysis 2](#_Toc70848760)

[Supplementary Table 1. Maximum EpiMatrix Z Score and Significant Z Score Count for all Predicted EBA175 Class II T Cell Epitope Clusters. 2](#_Toc70848761)

[Supplementary Table 2. Maximum EpiMatrix Z Score and Significant Z Score Count for all Predicted AMA1 Class II T Cell Epitope Clusters. 3](#_Toc70848762)

[Supplementary Table 3. Maximum EpiMatrix Z Score and Significant Z Score Count for all Predicted RIPR Class II T Cell Epitope Clusters. 4](#_Toc70848763)

[Supplementary Table 4. Maximum EpiMatrix Z Score and Significant Z Score Count for all Predicted CyRPA Class II T Cell Epitope Clusters. 5](#_Toc70848764)

[Supplementary Table 5. Maximum EpiMatrix Z Score and Significant Z Score Count for all Predicted RH5 Class II T Cell Epitope Clusters. 6](#_Toc70848765)

[Supplementary Table 6. Predicted EBA175 Class II Epitope Cross-Conservation with the Human Proteome as a Measure of Regulatory T Cell Response Potential. 7](#_Toc70848766)

[Supplementary Table 7. Predicted AMA1 Class II Epitope Cross-Conservation with the Human Proteome as a Measure of Regulatory T Cell Response Potential. 8](#_Toc70848767)

[Supplementary Table 8. Predicted RIPR Class II Epitope Cross-Conservation with the Human Proteome as a Measure of Regulatory T Cell Response Potential. 9](#_Toc70848768)

[Supplementary Table 9. Predicted CyRPA Class II Epitope Cross-Conservation with the Human Proteome as a Measure of Regulatory T Cell Response Potential. 10](#_Toc70848769)

[Supplementary Table 10. Predicted RH5 Class II Epitope Cross-Conservation with the Human Proteome as a Measure of Regulatory T Cell Response Potential. 11](#_Toc70848770)

[Supplementary Table 11. Peptide Synthesis Summary. 12](#_Toc70848771)

[Supplementary Table 12. In Vitro Class II HLA Binding Assay: Failed Peptide Listing. 12](#_Toc70848772)

[Supplementary Table 13. EBA175 Epitope In Vitro Binding Affinity (as IC_50_ in nM) for Class II HLA Alleles. 13](#_Toc70848773)

[Supplementary Table 14. AMA1 Epitope In Vitro Binding Affinity (as IC_50_ in nM) for Class II HLA Alleles. 14](#_Toc70848774)

[Supplementary Table 15. RIPR Epitope In Vitro Binding Affinity (as IC_50_ in nM) for Class II HLA Alleles. 15](#_Toc70848775)

[Supplementary Table 16. CyRPA Epitope In Vitro Binding Affinity (as IC_50_ in nM) for Class II HLA Alleles. 16](#_Toc70848776)

[Supplementary Table 17. RH5 Epitope In Vitro Binding Affinity (as IC_50_ in nM) for Class II HLA Alleles. 17](#_Toc70848777)

[Supplementary Table 18. In Vitro Class II HLA Binding Assay: Reference Peptide Sequences and Binding Affinity Color Key. 18](#_Toc70848778)

[Supplementary Table 19. Class II Epitope In Silico Predictions Demonstrated an Accuracy of 71% in Predicting Epitopes that Bind HLA-DR Alleles In Vitro. 19](#_Toc70848779)

[Supplementary Table 20. VAC063 Clinical Study Design – Dose Escalation. 20](#_Toc70848780)

[Supplementary Table 21. VAC057 Clinical Study Design – Dose Escalation. 20](#_Toc70848781)

[Supplementary Figure 1. VAC057 RH5 Ex Vivo Class II Epitope Cluster IFN-γ Recall Assay – Magnitude of Responses. 21](#_Toc70848782)

[4 Class I Epitope Prediction and Analysis 22](#_Toc70848783)

[Supplementary Materials and Methods 22](#_Toc70848784)

[Supplementary Table 22. In Vitro Class I HLA Binding Assay: Reference Peptide Sequences and Binding Affinity Color Key. 22](#_Toc70848785)

[Supplementary Table 23. EBA175 Class I HLA Allele In Vitro Binding Assay Data. 23](#_Toc70848786)

[Supplementary Table 24. AMA1 Class I HLA Allele In Vitro Binding Assay Data. 24](#_Toc70848787)

[Supplementary Table 25. RIPR Class I HLA Allele In Vitro Binding Assay Data. 26](#_Toc70848788)

[Supplementary Table 26. CyRPA Class I HLA Allele In Vitro Binding Assay Data. 27](#_Toc70848789)

[Supplementary Table 27. RH5 Class I HLA Allele In Vitro Binding Assay Data. 28](#_Toc70848790)

[Supplementary Table 28. Class I Epitope In Silico Predictions Demonstrated an Accuracy of 75% in Predicting Epitopes that Bind HLA-A and HLA-B Alleles In Vitro. 31](#_Toc70848791)

[Supplementary Table 29. Positive Donor Responses in the RH5 Class I HLA-A*0201 Ex Vivo IFN-γ T Cell Recall Assay. 31](#_Toc70848792)

[Supplementary Figure 2. Magnitude of Donor Responses in the RH5 Class I HLA-A*0201 Ex Vivo IFN-γ T Cell Recall Assay. 32](#_Toc70848793)

# Class II Epitope Prediction and Analysis

Supplementary Table 1. Maximum EpiMatrix Z Score and Significant Z Score Count for all Predicted EBA175 Class II T Cell Epitope Clusters.

EpiMatrix output from iVAX toolkit showing the highest Z score and total number of predicted epitopes (EpiMatrix hit count) for all EBA175 class II cluster across the set of HLA-DR alleles evaluated. EpiMatrix output identifies highly significant Z scores, in the top 1% (>2.31) and significant Z scores, in the top 5% (>1.63). Color coding indicates if the highest Z score falls in the top 1% (noted in dark blue) or the top 5% (noted in light blue). EpiMatrix hit counts for each HLA-DR are shown in parentheses.

|  | **DRB1*0101** | **DRB1*0301** | **DRB1*0401** | **DRB1*0701** | **DRB1*0801** | **DRB1*0901** | **DRB1*1101** | **DRB1*1301** | **DRB1*1501** |
| --- | --- | --- | --- | --- | --- | --- | --- | --- | --- |
| **Cluster ID** | **EPX Max Z (Total Hits)** | **EPX Max Z (Total Hits)** | **EPX Max Z (Total Hits)** | **EPX Max Z (Total Hits)** | **EPX Max Z (Total Hits)** | **EPX Max Z (Total Hits)** | **EPX Max Z (Total Hits)** | **EPX Max Z (Total Hits)** | **EPX Max Z (Total Hits)** |
| 4-28 | **1.72 (1)** | **2.36 (3)** | **1.97 (1)** | **1.91 (3)** | **2.60 (4)** | **2.45 (5)** | **2.05 (2)** | **2.61 (6)** | **2.92 (2)** |
| 64-86 | **1.64 (1)** | **2.50 (3)** | **1.96 (2)** | **1.86 (1)** | **2.63 (2)** | **1.92 (1)** | **2.27 (4)** | **2.19 (2)** | **1.52 (0)** |
| 101-122 | **2.53 (3)** | **1.85 (2)** | **3.05 (2)** | **2.49 (4)** | **2.43 (2)** | **2.55 (3)** | **1.93 (2)** | **2.49 (3)** | **2.82 (3)** |
| 114-140 | **2.69 (3)** | **2.12 (3)** | **2.26 (1)** | **2.56 (5)** | **1.85 (1)** | **1.71 (2)** | **2.18 (2)** | **2.18 (4)** | **2.06 (3)** |
| 183-206 | **1.66 (1)** | **1.75 (2)** | **2.56 (2)** | **2.19 (2)** | **2.78 (1)** | **2.28 (1)** | **1.83 (3)** | **2.07 (2)** | **1.76 (1)** |
| 205-220 | **1.38 (0)** | **2.17 (2)** | **1.12 (0)** | **1.32 (0)** | **2.29 (2)** | **2.14 (1)** | **2.15 (2)** | **2.02 (1)** | **1.68 (1)** |
| 261-279 | **2.51 (1)** | **2.08 (2)** | **2.56 (1)** | **1.65 (1)** | **1.95 (2)** | **2.29 (2)** | **1.90 (2)** | **1.56 (0)** | **1.82 (2)** |
| 293-310 | **2.09 (1)** | **2.74 (1)** | **2.22 (2)** | **1.73 (1)** | **1.67 (1)** | **1.68 (1)** | **2.16 (1)** | **1.70 (1)** | **1.24 (0)** |
| 328-344 | **1.91 (1)** | **2.97 (2)** | **3.14 (1)** | **1.63 (0)** | **1.42 (0)** | **1.24 (0)** | **2.40 (1)** | **1.74 (1)** | **1.75 (1)** |
| 516-538 | **1.21 (0)** | **2.14 (2)** | **1.70 (1)** | **1.91 (1)** | **3.51 (4)** | **1.56 (0)** | **1.82 (2)** | **1.78 (2)** | **1.86 (1)** |
| 556-579 | **2.50 (2)** | **2.19 (1)** | **2.77 (3)** | **2.41 (2)** | **1.86 (3)** | **1.66 (1)** | **2.20 (2)** | **1.97 (1)** | **1.50 (0)** |
| 687-707 | **2.10 (1)** | **2.22 (1)** | **2.18 (2)** | **1.77 (1)** | **2.26 (2)** | **2.29 (1)** | **1.76 (2)** | **1.72 (1)** | **2.24 (2)** |
| 699-721 | **2.17 (1)** | **2.14 (1)** | **1.97 (2)** | **2.14 (1)** | **2.35 (2)** | **2.28 (2)** | **1.86 (1)** | **1.98 (2)** | **1.60 (0)** |
| 747-763 | **1.82 (1)** | **2.06 (1)** | **2.57 (1)** | **1.92 (1)** | **1.75 (1)** | **0.95 (0)** | **2.40 (2)** | **1.18 (0)** | **1.94 (1)** |
| 1108-1124 | **1.53 (0)** | **1.92 (2)** | **1.93 (1)** | **1.66 (1)** | **2.48 (1)** | **1.70 (1)** | **1.76 (1)** | **2.22 (1)** | **1.30 (0)** |
| 1262-1276 | **2.88 (2)** | **1.89 (1)** | **3.03 (1)** | **1.46 (0)** | **2.50 (1)** | **2.11 (1)** | **3.05 (1)** | **1.85 (1)** | **2.59 (1)** |
| 1297-1321 | **2.49 (3)** | **1.91 (1)** | **2.25 (4)** | **2.39 (4)** | **2.26 (3)** | **2.12 (3)** | **2.37 (3)** | **1.18 (0)** | **2.39 (4)** |
| 1423-1449 | **2.09 (4)** | **1.98 (2)** | **2.29 (4)** | **2.17 (3)** | **1.75 (1)** | **2.47 (5)** | **2.49 (5)** | **2.05 (3)** | **2.21 (5)** |

Supplementary Table 2. Maximum EpiMatrix Z Score and Significant Z Score Count for all Predicted AMA1 Class II T Cell Epitope Clusters.

EpiMatrix output from iVAX toolkit showing the highest Z score and total number of predicted epitopes (EpiMatrix hit count) for all AMA1 class II cluster across the set of HLA-DR alleles evaluated. EpiMatrix output identifies highly significant Z scores, in the top 1% (>2.31) and significant Z scores, in the top 5% (>1.63). Color coding indicates if the highest Z score falls in the top 1% (noted in dark blue) or the top 5% (noted in light blue). EpiMatrix hit counts for each HLA-DR are shown in parentheses.

|  | **DRB1*0101** | **DRB1*0301** | **DRB1*0401** | **DRB1*0701** | **DRB1*0801** | **DRB1*0901** | **DRB1*1101** | **DRB1*1301** | **DRB1*1501** |
| --- | --- | --- | --- | --- | --- | --- | --- | --- | --- |
| **Cluster Address** | **EPX Max Z (Total Hits)** | **EPX Max Z (Total Hits)** | **EPX Max Z (Total Hits)** | **EPX Max Z (Total Hits)** | **EPX Max Z (Total Hits)** | **EPX Max Z (Total Hits)** | **EPX Max Z (Total Hits)** | **EPX Max Z (Total Hits)** | **EPX Max Z (Total Hits)** |
| 1-20 | **1.86 (2)** | **1.72 (1)** | **2.06 (1)** | **1.87 (3)** | **1.82 (1)** | **2.27 (2)** | **1.91 (2)** | **2.39 (2)** | **1.86 (3)** |
| 12-28 | **2.43 (2)** | **1.92 (2)** | **2.15 (1)** | **1.58 (0)** | **1.70 (1)** | **1.93 (1)** | **2.56 (2)** | **1.41 (0)** | **2.16 (1)** |
| 153-169 | **2.48 (2)** | **2.03 (2)** | **2.53 (2)** | **2.64 (2)** | **1.25 (0)** | **2.18 (2)** | **1.88 (1)** | **1.70 (2)** | **1.73 (1)** |
| 284-298 | **1.86 (1)** | **1.81 (1)** | **1.23 (0)** | **1.72 (1)** | **2.34 (2)** | **2.15 (1)** | **2.72 (1)** | **1.41 (0)** | **1.86 (2)** |
| 295-316 | **2.02 (2)** | **1.41 (0)** | **1.64 (1)** | **2.12 (2)** | **1.67 (2)** | **2.68 (1)** | **2.28 (2)** | **1.81 (1)** | **1.12 (0)** |
| 364-386 | **2.25 (1)** | **2.49 (2)** | **1.43 (0)** | **2.90 (1)** | **2.62 (2)** | **2.17 (3)** | **2.01 (2)** | **2.80 (2)** | **2.32 (2)** |
| 416-436 | **1.97 (2)** | **1.80 (1)** | **2.35 (3)** | **1.94 (4)** | **2.31 (2)** | **2.03 (3)** | **2.26 (2)** | **1.74 (1)** | **2.10 (1)** |
| 539-560 | **2.36 (4)** | **2.02 (3)** | **2.27 (4)** | **2.39 (4)** | **1.55 (0)** | **3.22 (4)** | **2.04 (4)** | **2.18 (3)** | **2.29 (2)** |
| 558-578 | **1.46 (0)** | **1.89 (3)** | **1.64 (1)** | **0.96 (0)** | **2.52 (6)** | **1.55 (0)** | **2.82 (4)** | **1.92 (2)** | **1.90 (1)** |

Supplementary Table 3. Maximum EpiMatrix Z Score and Significant Z Score Count for all Predicted RIPR Class II T Cell Epitope Clusters.

EpiMatrix output from iVAX toolkit showing the highest Z score and total number of predicted epitopes (EpiMatrix hit count) for all RIPR class II cluster across the set of HLA-DR alleles evaluated. EpiMatrix output identifies highly significant Z scores, in the top 1% (>2.31) and significant Z scores, in the top 5% (>1.63). Color coding indicates if the highest Z score falls in the top 1% (noted in dark blue) or the top 5% (noted in light blue). EpiMatrix hit counts for each HLA-DR are shown in parentheses.

|  | **DRB1*0101** | **DRB1*0301** | **DRB1*0401** | **DRB1*0701** | **DRB1*0801** | **DRB1*0901** | **DRB1*1101** | **DRB1*1301** | **DRB1*1501** |
| --- | --- | --- | --- | --- | --- | --- | --- | --- | --- |
| **Cluster Address** | **EPX Max Z (Total Hits)** | **EPX Max Z (Total Hits)** | **EPX Max Z (Total Hits)** | **EPX Max Z (Total Hits)** | **EPX Max Z (Total Hits)** | **EPX Max Z (Total Hits)** | **EPX Max Z (Total Hits)** | **EPX Max Z (Total Hits)** | **EPX Max Z (Total Hits)** |
| 1-25 | **2.58 (5)** | **2.29 (5)** | **3.03 (2)** | **2.97 (6)** | **2.71 (5)** | **3.02 (6)** | **2.91 (5)** | **3.26 (8)** | **3.25 (8)** |
| 61-84 | **2.47 (1)** | **2.86 (1)** | **2.19 (2)** | **2.14 (1)** | **2.27 (3)** | **1.95 (1)** | **2.34 (3)** | **1.70 (1)** | **1.82 (1)** |
| 140-163 | **2.78 (2)** | **2.46 (2)** | **2.32 (1)** | **3.35 (3)** | **2.49 (3)** | **3.09 (3)** | **2.36 (2)** | **2.54 (3)** | **2.59 (2)** |
| 181-203 | **3.31 (1)** | **1.89 (2)** | **2.79 (3)** | **3.22 (3)** | **2.27 (1)** | **2.00 (2)** | **2.12 (2)** | **2.18 (3)** | **2.46 (1)** |
| 231-253 | **2.43 (2)** | **1.59 (0)** | **2.09 (2)** | **2.60 (3)** | **1.60 (0)** | **2.00 (2)** | **2.02 (2)** | **2.35 (2)** | **2.14 (3)** |
| 406-432 | **1.88 (1)** | **2.18 (3)** | **2.50 (3)** | **2.15 (2)** | **2.89 (3)** | **2.32 (1)** | **2.26 (4)** | **2.03 (3)** | **2.39 (4)** |
| 469-492 | **1.97 (3)** | **2.85 (2)** | **2.34 (3)** | **1.97 (1)** | **2.07 (3)** | **2.74 (2)** | **1.94 (2)** | **1.79 (1)** | **2.04 (3)** |
| 486-508 | **2.01 (2)** | **2.86 (2)** | **1.76 (1)** | **1.62 (0)** | **1.46 (0)** | **2.27 (3)** | **1.77 (2)** | **2.71 (3)** | **1.71 (2)** |
| 502-523 | **2.26 (3)** | **2.82 (3)** | **1.57 (0)** | **2.57 (4)** | **2.45 (4)** | **1.93 (2)** | **2.86 (3)** | **2.34 (3)** | **2.21 (1)** |
| 526-547 | **1.94 (2)** | **1.24 (0)** | **2.07 (2)** | **2.83 (2)** | **1.31 (0)** | **1.71 (2)** | **1.68 (1)** | **1.91 (2)** | **1.44 (0)** |
| 552-577 | **2.97 (3)** | **1.78 (1)** | **2.03 (2)** | **3.30 (2)** | **2.48 (1)** | **2.50 (2)** | **2.20 (4)** | **2.71 (1)** | **2.83 (5)** |
| 594-614 | **2.51 (2)** | **1.82 (1)** | **2.88 (2)** | **2.54 (1)** | **2.19 (1)** | **2.24 (3)** | **2.12 (2)** | **2.75 (2)** | **2.96 (2)** |
| 627-644 | **2.04 (2)** | **1.43 (0)** | **1.36 (0)** | **2.32 (4)** | **1.38 (0)** | **2.27 (2)** | **1.58 (0)** | **1.89 (1)** | **1.90 (2)** |
| 662-676 | **2.37 (1)** | **1.87 (1)** | **1.52 (0)** | **2.93 (1)** | **2.36 (1)** | **2.38 (1)** | **1.91 (1)** | **1.51 (0)** | **1.58 (0)** |
| 704-718 | **2.76 (1)** | **0.84 (0)** | **1.78 (1)** | **3.06 (1)** | **1.83 (1)** | **1.61 (0)** | **1.85 (1)** | **1.25 (0)** | **1.85 (1)** |
| 742-756 | **3.00 (1)** | **2.14 (1)** | **2.17 (1)** | **2.24 (1)** | **0.81 (0)** | **1.33 (0)** | **0.77 (0)** | **1.88 (1)** | **1.23 (0)** |
| 802-817 | **2.67 (1)** | **2.22 (1)** | **1.81 (1)** | **2.77 (2)** | **1.37 (0)** | **1.68 (1)** | **1.43 (0)** | **1.58 (0)** | **1.45 (0)** |
| 1006-1031 | **2.92 (2)** | **1.75 (3)** | **2.87 (2)** | **2.61 (4)** | **2.73 (2)** | **2.02 (3)** | **2.95 (2)** | **1.76 (1)** | **2.11 (1)** |
| 1045-1065 | **1.87 (3)** | **1.78 (2)** | **1.62 (0)** | **2.50 (2)** | **3.00 (4)** | **2.01 (1)** | **2.70(2)** | **2.04 (1)** | **1.88 (2)** |

Supplementary Table 4. Maximum EpiMatrix Z Score and Significant Z Score Count for all Predicted CyRPA Class II T Cell Epitope Clusters.

EpiMatrix output from iVAX toolkit showing the highest Z score and total number of predicted epitopes (EpiMatrix hit count) for all CyRPA class II clusters across the set of HLA-DR alleles evaluated. EpiMatrix output identifies highly significant Z scores, in the top 1% (>2.31) and significant Z scores, in the top 5% (>1.63). Color coding indicates if the highest Z score falls in the top 1% (noted in dark blue) or the top 5% (noted in light blue). EpiMatrix hit counts for each HLA-DR are shown in parentheses.

|  | **DRB1*0101** | **DRB1*0301** | **DRB1*0401** | **DRB1*0701** | **DRB1*0801** | **DRB1*0901** | **DRB1*1101** | **DRB1*1301** | **DRB1*1501** |
| --- | --- | --- | --- | --- | --- | --- | --- | --- | --- |
| **Cluster Address** | **EPX Max Z (Total Hits)** | **EPX Max Z (Total Hits)** | **EPX Max Z (Total Hits)** | **EPX Max Z (Total Hits)** | **EPX Max Z (Total Hits)** | **EPX Max Z (Total Hits)** | **EPX Max Z (Total Hits)** | **EPX Max Z (Total Hits)** | **EPX Max Z (Total Hits)** |
| 2-30 | **2.79 (4)** | **2.35 (3)** | **2.37 (2)** | **3.10 (5)** | **2.46 (5)** | **2.83 (6)** | **2.19 (5)** | **2.51 (8)** | **2.44 (7)** |
| 31-53 | **2.27 (3)** | **2.23 (1)** | **2.15 (3)** | **1.99 (2)** | **2.27 (1)** | **1.99 (2)** | **2.22 (3)** | **2.29 (2)** | **1.95 (1)** |
| 151-172 | **1.66 (1)** | **2.94 (3)** | **2.16 (3)** | **2.39 (3)** | **2.92 (3)** | **2.90 (4)** | **2.79 (3)** | **2.62 (4)** | **2.76 (2)** |
| 222-242 | **2.81 (1)** | **1.63 (0)** | **2.83 (1)** | **1.97 (2)** | **2.75 (3)** | **1.54 (0)** | **3.11 (2)** | **2.07 (3)** | **2.17 (1)** |
| 239-258 | **1.68 (1)** | **2.08 (2)** | **1.90 (2)** | **1.98 (2)** | **1.45 (0)** | **2.19 (2)** | **1.32 (0)** | **2.17 (1)** | **1.57 (0)** |
| 261-284 | **2.00 (1)** | **3.37 (2)** | **2.44 (1)** | **2.09 (2)** | **2.82 (2)** | **1.72 (2)** | **1.95 (1)** | **2.22 (1)** | **1.68 (1)** |
| 301-318 | **3.00 (3)** | **2.52 (3)** | **2.76 (3)** | **3.08 (3)** | **2.29 (2)** | **2.12 (3)** | **2.29 (3)** | **2.23 (3)** | **2.11 (3)** |
| 330-355 | **2.37 (3)** | **2.63 (4)** | **2.01 (2)** | **3.04 (3)** | **1.81 (1)** | **2.94 (2)** | **1.68 (1)** | **1.88 (2)** | **2.24 (3)** |

Supplementary Table 5. Maximum EpiMatrix Z Score and Significant Z Score Count for all Predicted RH5 Class II T Cell Epitope Clusters.

EpiMatrix output from iVAX toolkit showing the highest Z score and total number of predicted epitopes (EpiMatrix hit count) for all RH5 class II cluster across the set of HLA-DR alleles evaluated. EpiMatrix output identifies highly significant Z scores, in the top 1% (>2.31) and significant Z scores, in the top 5% (>1.63). Color coding indicates if the highest Z score falls in the top 1% (noted in dark blue) or the top 5% (noted in light blue). EpiMatrix hit counts for each HLA-DR are shown in parentheses.

|  | **DRB1*0101** | **DRB1*0301** | **DRB1*0401** | **DRB1*0701** | **DRB1*0801** | **DRB1*0901** | **DRB1*1101** | **DRB1*1301** | **DRB1*1501** |
| --- | --- | --- | --- | --- | --- | --- | --- | --- | --- |
| **Cluster Address** | **EPX Max Z (Total Hits)** | **EPX Max Z (Total Hits)** | **EPX Max Z (Total Hits)** | **EPX Max Z (Total Hits)** | **EPX Max Z (Total Hits)** | **EPX Max Z (Total Hits)** | **EPX Max Z (Total Hits)** | **EPX Max Z (Total Hits)** | **EPX Max Z (Total Hits)** |
| 1-15 | **1.90 (1)** | **2.71 (3)** | **1.67 (1)** | **2.37 (3)** | **2.85 (3)** | **1.88 (2)** | **2.48 (2)** | **3.26 (3)** | **2.25 (2)** |
| 6-32 | **1.93 (3)** | **2.29 (3)** | **2.06 (4)** | **2.20 (6)** | **2.65 (5)** | **2.24 (3)** | **2.63 (5)** | **2.52 (4)** | **3.06 (5)** |
| 79-97 | **1.79 (2)** | **1.59 (0)** | **2.06 (1)** | **1.92 (2)** | **2.11 (2)** | **2.80 (2)** | **2.02 (1)** | **2.42 (1)** | **2.39 (1)** |
| 92-110 | **2.63 (2)** | **2.57 (2)** | **2.88 (2)** | **2.71 (2)** | **2.30 (1)** | **3.11 (2)** | **2.32 (2)** | **2.57 (2)** | **2.11 (1)** |
| 144-166 | **2.69 (2)** | **2.18 (3)** | **2.24 (3)** | **3.04 (3)** | **2.25 (2)** | **2.46 (2)** | **2.20 (1)** | **2.53 (5)** | **2.54 (2)** |
| 179-205 | **2.93 (3)** | **2.60 (1)** | **2.58 (3)** | **3.23 (4)** | **2.13 (5)** | **2.60 (4)** | **3.05 (3)** | **2.38 (7)** | **2.95 (3)** |
| 224-246 | **1.41 (0)** | **2.38 (1)** | **1.48 (0)** | **1.78 (1)** | **2.76 (2)** | **1.92 (1)** | **1.83 (2)** | **2.10 (1)** | **1.80 (1)** |
| 304-326 | **1.94 (1)** | **1.75 (1)** | **1.99 (1)** | **1.70 (2)** | **2.94 (2)** | **1.66 (1)** | **1.98 (2)** | **2.38 (1)** | **1.28 (0)** |
| 360-382 | **3.66 (2)** | **2.88 (2)** | **3.15 (2)** | **3.65 (2)** | **2.91 (6)** | **2.82 (3)** | **3.16 (5)** | **2.99 (3)** | **3.17 (3)** |
| 382-408 | **1.89 (3)** | **1.79 (2)** | **1.74 (1)** | **1.95 (4)** | **2.87 (3)** | **1.95 (3)** | **2.58 (4)** | **2.62 (4)** | **2.13 (4)** |
| 453-473 | **1.76 (1)** | **2.52 (2)** | **2.03 (2)** | **1.89 (2)** | **1.82 (1)** | **1.50 (0)** | **1.78 (1)** | **1.58 (0)** | **2.38 (1)** |
| 480-505 | **2.78 (2)** | **1.85 (1)** | **2.24 (4)** | **2.40 (4)** | **2.42 (3)** | **2.02 (3)** | **1.96 (2)** | **2.19 (4)** | **1.96 (2)** |
| 512-526 | **2.57 (1)** | **2.09 (1)** | **3.16 (2)** | **2.59 (1)** | **2.79 (4)** | **2.20 (2)** | **3.32 (2)** | **2.21 (2)** | **1.93 (1)** |

Supplementary Table 6. Predicted EBA175 Class II Epitope Cross-Conservation with the Human Proteome as a Measure of Regulatory T Cell Response Potential.

EpiMatrix (EPX) cluster scores (a measure of immune potential) and JanusMatrix (JMX) cluster scores (a measure of Treg potential) are shown for the clusters of in silico predicted class II epitopes within EBA175. A JMX cluster score >2.00 denotes increased human-ness flags. The number of human matches indicates the number of human proteins containing synonymous HLA-DR binding frames with matching TCR-binding face residues as compared to those in the EBA175 clusters. For each HLA-DR allele, the match count is shown (i.e., total number of matched, human homology Z scores ≥1.64 for each allele from JMX outputs). Sequences include the binding core (bold) and flanking residues.

| **Cluster Address** | **Sequence** | **EPX Cluster Score** | **Number HUMAN Matches** | **JMX Cluster Score** | **JMX Total Z Score Hit Count (≥1.64)** | | | | | | | | |
| --- | --- | --- | --- | --- | --- | --- | --- | --- | --- | --- | --- | --- | --- |
|  |  |  |  |  | **DRB1 *0101** | **DRB1 *0301** | **DRB1 *0401** | **DRB1 *0701** | **DRB1 *0801** | **DRB1 *0901** | **DRB1 *1101** | **DRB1 *1301** | **DRB1 *1501** |
| 4-28 | NIS**IYFFASFFVLYFAKARNEY**DIK | 40.44 | 8 | 0.37 | 0 | 1 | 0 | 0 | 1 | 2 | 0 | 2 | 0 |
| 64-86 | ENK**LDILNNSKFNKRWKSYG**TPD | 19.5 | 2 | 0.13 | 0 | 0 | 1 | 0 | 0 | 0 | 1 | 0 | 0 |
| 101-122 | EEM**FNNNYQSFLSTSSLIK**QNK | 45.51 | 20 | 2.58 | 3 | 2 | 6 | 9 | 8 | 5 | 8 | 11 | 10 |
| 114-140 | TSS**LIKQNKYVPINAVRVSRILSF**LDS | 34.49 | 17 | 1.21 | 3 | 6 | 0 | 5 | 0 | 0 | 1 | 7 | 7 |
| 183-206 | DRR**IQLCIVNLSIIKTYTKET**MKD | 18.66 | 11 | 1.53 | 0 | 6 | 4 | 1 | 7 | 0 | 5 | 0 | 0 |
| 205-220 | KDH**FIEASKKESQ**LLL | 16.25 | 2 | 0.33 | 0 | 0 | 0 | 0 | 2 | 0 | 1 | 0 | 0 |
| 261-279 | ENK**IQEVFKGAHGEIS**EHK | 21.33 | 3 | 0.69 | 1 | 1 | 1 | 1 | 1 | 1 | 1 | 0 | 2 |
| 293-310 | EKL**WEAMLSEHKNNI**NNC | 10.55 | 2 | 0.22 | 1 | 0 | 1 | 0 | 0 | 0 | 0 | 0 | 0 |
| 328-344 | HGE**FLLERDNRSKL**PKS | 13.47 | 6 | 2.14 | 1 | 3 | 3 | 0 | 0 | 0 | 3 | 1 | 4 |
| 516-538 | ILA**IAIYESRILKRKYKNKD**DKE | 12.17 | 12 | 1 | 0 | 1 | 0 | 0 | 8 | 0 | 2 | 2 | 0 |
| 556-579 | GTD**YWNDLSNRKLVGKINTNS**NYV | 16.43 | 4 | 0.33 | 2 | 0 | 0 | 0 | 1 | 0 | 2 | 0 | 0 |
| 687-707 | YQE**YQKGNNYKMYSEFKS**IKP | 15.12 | 3 | 0.69 | 1 | 1 | 2 | 1 | 0 | 1 | 1 | 0 | 2 |
| 699-721 | YSE**FKSIKPEVYLKKYSEKC**SNL | 12.12 | 9 | 1.58 | 2 | 1 | 2 | 2 | 3 | 4 | 2 | 3 | 0 |
| 747-763 | DVP**ISIIRNNEQTS**QEA | 13.47 | 1 | 0.25 | 1 | 0 | 0 | 1 | 0 | 0 | 0 | 0 | 0 |
| 1108-1124 | YED**IVLKSHMNRES**DDG | 12.66 | 0 | 0 | 0 | 0 | 0 | 0 | 0 | 0 | 0 | 0 | 0 |
| 1262-1276 | KHG**FHTMNNLHG**DGV | 18.98 | 3 | 0.89 | 1 | 1 | 0 | 0 | 1 | 1 | 0 | 3 | 1 |
| 1297-1321 | SGN**VLNMRSNNNNFNNIPSRYN**LYD | 41.01 | 4 | 0.36 | 0 | 0 | 2 | 0 | 1 | 0 | 3 | 0 | 0 |
| 1423-1449 | MPY**YAGAGVLFIILVILGASQAKY**QSS | 48.34 | 33 | 1.5 | 10 | 0 | 10 | 4 | 0 | 4 | 11 | 3 | 6 |

Supplementary Table 7. Predicted AMA1 Class II Epitope Cross-Conservation with the Human Proteome as a Measure of Regulatory T Cell Response Potential.

EpiMatrix (EPX) cluster scores (a measure of immune potential) and JanusMatrix (JMX) cluster scores (a measure of Treg potential) are shown for the clusters of in silico predicted class II epitopes within AMA1. A JMX cluster score >2.00 denotes increased human-ness flags. The number of human matches indicates the number of human proteins containing synonymous HLA-DR binding frames with matching TCR-binding face residues as compared to those in the AMA1 clusters. For each HLA-DR allele, the match count is shown (i.e., total number of matched, human homology Z scores ≥1.64 for each allele from JMX outputs). Sequences include the binding core (bold) and flanking residues.

| **Cluster Address** | **Sequence** | **EPX Cluster Score** | **Number HUMAN Matches** | **JMX Cluster Score** | **JMX Total Z Score Hit Count (≥1.64)** | | | | | | | | |
| --- | --- | --- | --- | --- | --- | --- | --- | --- | --- | --- | --- | --- | --- |
|  |  |  |  |  | **DRB1 *0101** | **DRB1 *0301** | **DRB1 *0401** | **DRB1 *0701** | **DRB1 *0801** | **DRB1 *0901** | **DRB1 *1101** | **DRB1 *1301** | **DRB1 *1501** |
| 1-20 | **MRKLYCVLLLSAFEFTY**MIN | 23.23 | 15 | 1.18 | 1 | 0 | 0 | 2 | 5 | 3 | 1 | 3 | 5 |
| 12-28 | AFE**FTYMINFGRGQ**NYW | 16.08 | 0 | 0 | 0 | 0 | 0 | 0 | 0 | 0 | 0 | 0 | 0 |
| 153-169 | GKG**IIIENSNTTFL**TPV | 25.61 | 2 | 0.14 | 0 | 1 | 0 | 0 | 0 | 0 | 0 | 1 | 0 |
| 284-298 | FQN**YTYLSKNVV**DNW | 13.39 | 2 | 0.22 | 0 | 0 | 0 | 0 | 0 | 0 | 0 | 0 | 2 |
| 295-316 | VDN**WEKVCPRKNLQNAKFG**LWV | 11.62 | 6 | 0.64 | 1 | 0 | 1 | 2 | 1 | 0 | 1 | 1 | 0 |
| 364-386 | KEG**FKNKNASMIKSAFLPTG**AFK | 24.86 | 3 | 0.73 | 0 | 2 | 0 | 0 | 3 | 1 | 3 | 2 | 0 |
| 416-436 | PTC**LINNSSYIATTALSH**PIE | 28.63 | 15 | 1.05 | 3 | 0 | 3 | 6 | 1 | 6 | 0 | 1 | 0 |
| 539-560 | KPT**YDKMKIIIASSAAVAV**LAT | 51.66 | 17 | 1.39 | 6 | 5 | 5 | 1 | 0 | 14 | 3 | 4 | 1 |
| 558-578 | LAT**ILMVYLYKRKGNAEK**YDK | 27.98 | 5 | 0.29 | 0 | 1 | 0 | 0 | 5 | 1 | 1 | 0 | 1 |

Supplementary Table 8. Predicted RIPR Class II Epitope Cross-Conservation with the Human Proteome as a Measure of Regulatory T Cell Response Potential.

EpiMatrix (EPX) cluster scores (a measure of immune potential) and JanusMatrix (JMX) cluster scores (a measure of Treg potential) are shown for clusters of in silico predicted class II epitopes within RIPR. A JMX cluster score >2.00 denotes increased human-ness flags. The number of human matches indicates the number of human proteins containing synonymous HLA-DR binding frames with matching TCR-binding face residues as compared to those in the RIPR clusters. For each HLA-DR allele, the match count is shown (i.e., total number of matched, human homology Z scores ≥1.64 for each allele from JMX outputs). Sequences include the binding core (bold) and flanking residues.

| **Cluster Address** | **Sequence** | **EPX Cluster Score** | **Number HUMAN Matches** | **JMX Cluster Score** | **JMX Total Z Score Hit Count (≥1.64)** | | | | | | | | |
| --- | --- | --- | --- | --- | --- | --- | --- | --- | --- | --- | --- | --- | --- |
|  |  |  |  |  | **DRB1 *0101** | **DRB1 *0301** | **DRB1 *0401** | **DRB1 *0701** | **DRB1 *0801** | **DRB1 *0901** | **DRB1 *1101** | **DRB1 *1301** | **DRB1 *1501** |
| 1-25 | M**FRIFFTLLIIILIKKTSAIDL**IEG | 99.43 | 27 | 1.28 | 4 | 9 | 5 | 5 | 9 | 7 | 3 | 13 | 10 |
| 61-84 | DYA**YLNKYVYTILNRDSTEKI**KTF | 17.88 | 5 | 0.5 | 1 | 0 | 0 | 1 | 2 | 1 | 0 | 1 | 1 |
| 140-163 | CAH**FNSTHIIIYYISQPLLLE**PHV | 37.37 | 9 | 1.1 | 1 | 5 | 1 | 1 | 3 | 1 | 4 | 6 | 1 |
| 181-203 | QGM**YISLRSVHVHTHNAILQ**QET | 29.59 | 5 | 0.39 | 0 | 1 | 1 | 3 | 0 | 1 | 0 | 1 | 0 |
| 231-253 | LTH**YLFFINIQYQCISPLNL**QEN | 25.68 | 3 | 0.19 | 0 | 0 | 1 | 2 | 0 | 0 | 0 | 0 | 0 |
| 406-432 | SIE**VINAYVSCYRVSFNLNKLKYV**TES | 30.25 | 4 | 0.21 | 0 | 0 | 2 | 0 | 2 | 0 | 1 | 0 | 0 |
| 469-492 | KYD**YLCVFNNQNITSDKNSHL**HSN | 30.53 | 5 | 0.45 | 2 | 3 | 2 | 0 | 0 | 2 | 0 | 0 | 0 |
| 486-508 | NSH**LHSNIPSLYNSSILPDI**NKS | 19.26 | 11 | 2 | 2 | 6 | 3 | 0 | 0 | 7 | 4 | 8 | 0 |
| 502-523 | LPD**INKSKFHLISRNSRTN**QYP | 36.32 | 11 | 1 | 0 | 5 | 0 | 5 | 3 | 4 | 3 | 3 | 0 |
| 526-547 | NIS**MLEIQNEISSHNSNQF**STD | 13.66 | 1 | 0.09 | 0 | 0 | 0 | 1 | 0 | 0 | 0 | 0 | 0 |
| 552-577 | SNN**INNMNIKKVEIFRSRFSSKL**QCQ | 31.94 | 9 | 1.43 | 4 | 2 | 2 | 4 | 2 | 6 | 5 | 2 | 3 |
| 594-614 | CND**LLLTNSLKSYCNDLS**ECD | 28.47 | 31 | 7.31 | 11 | 10 | 15 | 11 | 11 | 9 | 16 | 17 | 18 |
| 627-644 | NDQ**YLFVSYSCSNLC**NKC | 17.33 | 4 | 0.36 | 0 | 0 | 0 | 1 | 0 | 2 | 0 | 0 | 1 |
| 662-676 | DNP**YISKYGNKL**CER | 12.88 | 0 | 0 | 0 | 0 | 0 | 0 | 0 | 0 | 0 | 0 | 0 |
| 704-718 | EEG**YKNVKGKCV**PDN | 10.37 | 0 | 0 | 0 | 0 | 0 | 0 | 0 | 0 | 0 | 0 | 0 |
| 742-756 | SER**FVLENGVCI**CAN | 10.5 | 2 | 0.8 | 2 | 0 | 0 | 2 | 0 | 0 | 0 | 0 | 0 |
| 802-817 | KEH**YYRSSRGECI**LND | 11.01 | 2 | 0.5 | 2 | 0 | 1 | 3 | 0 | 1 | 0 | 0 | 0 |
| 1006-1031 | MIE**FSYIYNQIIWKINNSKESYV**FYY | 32.08 | 3 | 0.3 | 0 | 1 | 0 | 2 | 1 | 1 | 1 | 0 | 0 |
| 1045-1065 | NEI**FHTIIYLKKKIGNSV**IYD | 26.67 | 7 | 0.65 | 0 | 1 | 0 | 3 | 3 | 2 | 1 | 0 | 0 |

Supplementary Table 9. Predicted CyRPA Class II Epitope Cross-Conservation with the Human Proteome as a Measure of Regulatory T Cell Response Potential.

EpiMatrix (EPX) cluster scores (a measure of immune potential) and JanusMatrix (JMX) cluster scores (a measure of Treg potential) are shown for the clusters of in silico predicted class II epitopes within CyRPA. A JMX cluster score >2.00 denotes increased human-ness flags. The number of human matches indicates the number of human proteins containing synonymous HLA-DR binding frames with matching TCR-binding face residues as compared to those in the CyRPA clusters. For each HLA-DR allele, the match count is shown (i.e., total number of matched, human homology Z scores ≥1.64 for each allele from JMX outputs). Sequences include the binding core (bold) and flanking residues.

| **Cluster Address** | **Sequence** | **EPX Cluster Score** | **Number HUMAN Matches** | **JMX Cluster Score** | **JMX Total Z Score Hit Count (≥1.64)** | | | | | | | | |
| --- | --- | --- | --- | --- | --- | --- | --- | --- | --- | --- | --- | --- | --- |
|  |  |  |  |  | **DRB1 *0101** | **DRB1 *0301** | **DRB1 *0401** | **DRB1 *0701** | **DRB1 *0801** | **DRB1 *0901** | **DRB1 *1101** | **DRB1 *1301** | **DRB1 *1501** |
| 2-30 | IIP**FHKKFISFFQIVLVVLLLCRSIN**CDS | 65.22 | 75 | 3.67 | 16 | 5 | 12 | 13 | 8 | 32 | 37 | 11 | 32 |
| 31-53 | RHV**FIRTELSFIKNNVPCIR**DMF | 25.53 | 5 | 0.83 | 2 | 2 | 2 | 2 | 1 | 1 | 4 | 2 | 0 |
| 151-172 | ISD**YILKDKLLSSYVSLPL**KIE | 43.33 | 66 | 5.04 | 0 | 21 | 17 | 14 | 16 | 11 | 18 | 33 | 2 |
| 222-242 | GVQ**YFFLRPYISKNDLSF**HFY | 20.7 | 7 | 0.62 | 0 | 0 | 0 | 1 | 1 | 0 | 3 | 3 | 0 |
| 239-258 | FHF**YVGDNINNVKNVNF**IEC | 12.34 | 4 | 0.7 | 0 | 2 | 2 | 1 | 0 | 2 | 0 | 1 | 0 |
| 261-284 | EKD**LEFVCSNRDFLKDNKVLQ**DVS | 18.4 | 11 | 2.23 | 0 | 8 | 5 | 0 | 3 | 4 | 1 | 8 | 0 |
| 301-318 | AEC**YIFFNNENSILI**KPE | 53.91 | 5 | 0.23 | 2 | 0 | 1 | 0 | 0 | 0 | 0 | 0 | 1 |
| 330-355 | GTF**VKIDENRTLFIYSSSQGIYN**IHT | 28.74 | 6 | 0.33 | 0 | 2 | 1 | 0 | 1 | 0 | 0 | 1 | 2 |

Supplementary Table 10. Predicted RH5 Class II Epitope Cross-Conservation with the Human Proteome as a Measure of Regulatory T Cell Response Potential.

EpiMatrix (EPX) cluster scores (a measure of immune potential) and JanusMatrix (JMX) cluster scores (a measure of Treg potential) are shown for the clusters of in silico predicted class II epitopes within RH5. A JMX cluster score >2.00 denotes increased human-ness flags. The number of human matches indicates the number of human proteins containing synonymous HLA-DR binding frames with matching TCR-binding face residues as compared to those in the RH5 clusters. For each HLA-DR allele, the match count is shown (i.e., total number of matched, human homology Z scores ≥1.64 for each allele from JMX outputs). Sequences include the binding core (bold) and flanking residues.

| **Cluster Address** | **Sequence** | **EPX Cluster Score** | **Number HUMAN Matches** | **JMX Cluster Score** | **JMX Total Z Score Hit Count (≥1.64)** | | | | | | | | |
| --- | --- | --- | --- | --- | --- | --- | --- | --- | --- | --- | --- | --- | --- |
|  |  |  |  |  | **DRB1 *0101** | **DRB1 *0301** | **DRB1 *0401** | **DRB1 *0701** | **DRB1 *0801** | **DRB1 *0901** | **DRB1 *1101** | **DRB1 *1301** | **DRB1 *1501** |
| 1-15 | **MIRIKKKLILTI**IYI | 37.83 | 18 | 2.40 | 1 | 6 | 3 | 10 | 8 | 5 | 4 | 9 | 2 |
| 6-32 | KKL**ILTIIYIHLFILNRLSFENAI**KKT | 59.65 | 18 | 0.95 | 3 | 3 | 4 | 5 | 1 | 1 | 10 | 2 | 11 |
| 79-97 | HST**YIKSYLNTNVNDG**LKY | 18.6 | 1 | 0.25 | 1 | 0 | 1 | 0 | 1 | 0 | 0 | 1 | 1 |
| 92-110 | NDG**LKYLFIPSHNSFI**KKY | 31.73 | 2 | 0.13 | 0 | 0 | 0 | 0 | 0 | 0 | 1 | 1 | 0 |
| 144-166 | FLQ**YHFKELSNYNIANSIDI**LQE | 36.38 | 5 | 0.26 | 0 | 1 | 1 | 3 | 0 | 0 | 0 | 1 | 0 |
| 179-205 | YTF**LDYYKHLSYNSIYHKSSTYGK**CIA | 53.65 | 7 | 0.52 | 1 | 1 | 1 | 2 | 2 | 3 | 2 | 1 | 3 |
| 224-246 | CND**IKNDLIATIKKLEHPYD**INN | 10.36 | 3 | 0.44 | 0 | 0 | 0 | 1 | 1 | 1 | 0 | 1 | 0 |
| 304-326 | MDE**YNTKKKKLIKCIKNHEN**DFN | 14.34 | 8 | 1.55 | 0 | 0 | 0 | 6 | 4 | 2 | 3 | 3 | 0 |
| 360-382 | YDE**YIHKLILSVKSKNLNKD**LSD | 49.71 | 24 | 1.93 | 4 | 4 | 3 | 4 | 10 | 7 | 9 | 9 | 7 |
| 382-408 | DMT**NILQQSELLLTNLNKKMGSYI**YID | 41.96 | 47 | 3.29 | 6 | 5 | 5 | 15 | 9 | 4 | 14 | 14 | 20 |
| 453-473 | DEL**LKRILDMSNEYSLFI**TSD | 11.03 | 6 | 0.8 | 0 | 0 | 0 | 0 | 3 | 0 | 2 | 0 | 3 |
| 480-505 | YNT**FYSKEKHLNNIFHHLIYVLQ**MKF | 34.77 | 9 | 0.36 | 0 | 0 | 2 | 4 | 0 | 1 | 0 | 3 | 0 |
| 512-526 | MEY**FQTYKKNKPLTQ** | 28.98 | 8 | 1.75 | 1 | 3 | 4 | 2 | 4 | 2 | 4 | 4 | 4 |

Supplementary Table 11. Peptide Synthesis Summary.

|  | **Clusters (N)** | | |  |
| --- | --- | --- | --- | --- |
| **Protein Name** | **Total** | **Attempted Synthesis** | **Successful Synthesis** | **Peptides (N)** |
| **EBA175** | **18** | **12** | **12** | **14^a^** |
| **AMA1** | **9** | **8** | **6** | **8^b^** |
| **RIPR** | **19** | **18** | **10** | **10** |
| **CyRPA** | **8** | **6** | **2** | **2** |
| **RH5** | **13** | **10** | **10** | **12^a^** |
| **Total** | **67** | **54** | **40** | **46** |

^a^For two clusters, two peptides were synthesized for each cluster to accommodate a long cluster sequence. ^b^For one cluster, long peptide synthesis was successful and two shorter peptides (with the cluster sequence divided between them) were also synthesized for the same cluster.

Supplementary Table 12. In Vitro Class II HLA Binding Assay: Failed Peptide Listing.

Sequences that were submitted for peptide synthesis but that failed due to problems with the synthesis or purification steps are listed below. Note that peptide name relates to the consecutively numbered cluster which the peptide represents in each of the proteins.

| **Peptide Name** | **Peptide Address** | **Peptide Sequence** |
| --- | --- | --- |
| AMA1_003 | 153-169 | KKGIIIENSNTTFLTPV** |
| AMA1_008 | 541-560 | TYDKMKIIIASSAAVAVLAT* |
| CYRPA_002 | 31-53 | RHVFIRTELSFIKNNVPCIRDMF |
| CYRPA_004 | 222-242 | GVQYFFLRPYISKNDLSFHFY |
| CYRPA_006 | 261-284 | EKDLEFVCSNRDFLKDNKVLQDVS |
| CYRPA_007 | 301-319 | AECYIFFNNENSILIKPEK** |
| RIPR_001 | 1-24 | MFRIFFTLLIIILIKKTSAIDLIE* |
| RIPR_003 | 146-164 | THIIIYYISQPLLLEPHV* |
| RIPR_004 | 182-201 | GMYISLRSVHVHTHNAILQQ* |
| RIPR_005 | 231-253 | LTHYLFFINIQYQCISPLNLQEN |
| RIPR_010 | 526-547 | NISMLEIQNEISSHNSNQFSTD |
| RIPR_013 | 627-644 | NDQYLFVSYSCSNLCNKC |
| RIPR_016 | 742-756 | SERFVLENGVCICAN |
| RIPR_018 | 1007-1032 | IEFSYIYNQIIWKINNSKESYVFYYD*^,^** |

*Peptide trimmed from original ClustiMer output. **Charge added to facilitate peptide synthesis and purification.

Supplementary Table 13. EBA175 Epitope In Vitro Binding Affinity (as IC_50_ in nM) for Class II HLA Alleles.

A subset of predicted EBA175 class II epitope clusters were synthesized as peptides and assessed for HLA-DR binding in vitro utilizing a binding inhibition assay. Note that peptide name relates to the consecutively numbered cluster which the peptide represents in EBA175. The A and B designations are used when two peptides include sequences from the same cluster.

| **Peptide Name** | **Peptide Address** | **Peptide Sequence** | **DRB1*0101** | **DRB1*0303** | **DRB1*0401** | **DRB1*0701** | **DRB1*0801** | **DRB1*1101** | **DRB1*1301** | **DRB1*1501** |
| --- | --- | --- | --- | --- | --- | --- | --- | --- | --- | --- |
|  |  |  | **IC_50_** | **IC_50_** | **IC_50_** | **IC_50_** | **IC_50_** | **IC_50_** | **IC_50_** | **IC_50_** |
| EBA175_001A | 4-20 | NISIYFFASFFVLYFAK-amide* | **896** | **1,205** | **719** | **983** | **9,344** | **357** | **1,188** | **356** |
| EBA175_001B | 11-28 | Ac-ASFFVLYFAKARNEYDIK-amide* | **678** | **1,566** | **2,267** | **421** | **9,879** | **520** | **2,239** | **265** |
| EBA175_002 | 64-80 | Ac-ENKLDILNNSKFNKRWK-amide* | **11,867** | **14,275** | **NB** | **145,943** | **149,150** | **161,328** | **14,207** | **13,938** |
| EBA175_004 | 119-140 | Ac-KQNKYVPINAVRVSRILSFLDS-amide* | **4** | **392** | **18,214** | **18** | **6,117** | **396** | **1,365** | **2,073** |
| EBA175_006 | 206-219 | Ac-DHFIEASKKESQLL-amide* | **6,626** | **1,349** | **NB** | **102,170** | **1,764** | **2,866** | **74,550** | **159,823** |
| EBA175_008 | 293-309 | Ac-EKLWEAMLSEHKNNINN-amide* | **5,613** | **141,922** | **548,430** | **256,351** | **115,917** | **NB** | **NB** | **80,638** |
| EBA175_010 | 516-538 | Ac-ILAIAIYESRILKRKYKNKDDKE-amide | **270** | **419** | **152,768** | **6,269** | **17,551** | **44,194** | **4,413** | **35** |
| EBA175_011 | 556-575 | Ac-GTDYWNDLSNRKLVGKINTN-amide* | **462** | **1,718,813** | **52,684** | **140,185** | **NB** | **28,619** | **NB** | **54,192** |
| EBA175_012 | 687-707 | Ac-YQEYQKGNNYKMYSEFKSIK-amide* | **226** | **29,587** | **117,376** | **14,023** | **33,200** | **120,334** | **109,744** | **29,445** |
| EBA175_014 | 747-763 | Ac-DVPISIIRNNEQTSQEA-amide | **6,148** | **3,867** | **3,807** | **13,338** | **18,732** | **16,706** | **14,139** | **3,091** |
| EBA175_015 | 1108-1124 | Ac-YEDIVLKSHMNRESDD-amide* | **19,744** | **NB** | **455,802** | **NB** | **111,979** | **271,905** | **4,919** | **36,008** |
| EBA175_016 | 1262-1278 | Ac-KHGFHTMNNLHGDGVSE-amide* | **160** | **658,996** | **458** | **165,834** | **106,395** | **8,623** | **NB** | **79,050** |
| EBA175_017A | 1297-1313 | SGNVLNMRSNNNNFNNI-amide* | **814** | **1,985** | **2,559** | **1,097** | **13,865** | **3,126** | **7,416** | **2,722** |
| EBA175_017B | 1304-1321 | Ac-RSNNNNFNNIPSRYNLYD-amide* | **430** | **NB** | **2,289,874** | **121,661** | **6,334,533** | **674,841** | **NB** | **23,609** |

*Peptide trimmed from original ClustiMer output.

Supplementary Table 14. AMA1 Epitope In Vitro Binding Affinity (as IC_50_ in nM) for Class II HLA Alleles.

A subset of predicted AMA1 class II epitope clusters were synthesized as peptides and assessed for HLA-DR binding in vitro utilizing a binding inhibition assay. Note that peptide name relates to the consecutively numbered cluster which the peptide represents in AMA1. The A and B designations are used when two peptides include sequences from the same cluster. The peptide AMA1_006C represents the full cluster sequence.

| **Peptide Name** | **Peptide Address** | **Peptide Sequence** | **DRB1*0101** | **DRB1*0303** | **DRB1*0401** | **DRB1*0701** | **DRB1*0801** | **DRB1*1101** | **DRB1*1301** | **DRB1*1501** |
| --- | --- | --- | --- | --- | --- | --- | --- | --- | --- | --- |
|  |  |  | **IC_50_** | **IC_50_** | **IC_50_** | **IC_50_** | **IC_50_** | **IC_50_** | **IC_50_** | **IC_50_** |
| AMA1_001 | 1-17 | MRKLYCVLLLSAFEFTY-amide* | **394** | **1,038** | **1,465** | **206** | **17,265** | **320** | **1,787** | **751** |
| AMA1_002 | 12-29 | AFEFTYMINFGRGQNYWE-amide** | **117** | **87** | **45** | **113** | **239** | **236** | **723** | **76** |
| AMA1_004 | 284-299 | Ac-FQNYTYLSKNVVDNWE-amide** | **16** | **11,205** | **9,689** | **9,480** | **10** | **567** | **69,974** | **248** |
| AMA1_006A | 364-377 | Ac-KEGFKNKNASMIKS-amide* | **3,941** | **NB** | **NB** | **NB** | **3,735** | **NB** | **NB** | **NB** |
| AMA1_006B | 372-386 | ASMIKSAFLPTGAFK-amide* | **3,224** | **126,394** | **532,757** | **1,516** | **532,627** | **149,572** | **NB** | **14,458** |
| AMA1_006C | 364-386 | Ac-KEGFKNKNASMIKSAFLPTGAFK-amide | **1,200** | **138,552** | **241,112** | **1,291** | **50,391** | **228,849** | **NB** | **2,044** |
| AMA1_007 | 417-436 | TCLINNSSYIATTALSHPIE-amide* | **54** | **304** | **208** | **99** | **13,530** | **577** | **1,442** | **679** |
| AMA1_009 | 558-578 | Ac-LATILMVYLYKRKGNAEKYDK-amide | **10,013** | **3,033** | **11,077** | **3,402** | **7,985** | **165** | **4,398** | **2,435** |

*Peptide trimmed from original ClustiMer output. **Charge added to facilitate peptide synthesis and purification.

Supplementary Table 15. RIPR Epitope In Vitro Binding Affinity (as IC_50_ in nM) for Class II HLA Alleles.

A subset of predicted RIPR class II epitope clusters were synthesized as peptides and assessed for HLA-DR binding in vitro utilizing a binding inhibition assay. Note that peptide name relates to the consecutively numbered cluster which the peptide represents in RIPR.

| **Peptide Name** | **Peptide Address** | **Peptide Sequence** | **DRB1*0101** | **DRB1*0303** | **DRB1*0401** | **DRB1*0701** | **DRB1*0801** | **DRB1*1101** | **DRB1*1301** | **DRB1*1501** |
| --- | --- | --- | --- | --- | --- | --- | --- | --- | --- | --- |
|  |  |  | **IC_50_** | **IC_50_** | **IC_50_** | **IC_50_** | **IC_50_** | **IC_50_** | **IC_50_** | **IC_50_** |
| RIPR_002 | 61-84 | Ac-DYAYLNKYVYTILNRDSTEKIKTF-amide | **152** | **1,089** | **16,742** | **6,910** | **16,129** | **1,467** | **50,397** | **201** |
| RIPR_006 | 414-432 | Ac-VSCYRVSFNLNKLKYVTES-amide* | **446** | **10,251** | **8,778** | **952** | **11,288** | **9,317** | **11,514** | **6,961** |
| RIPR_007 | 470-486 | YDYLCVFNNQNITSDKN-amide* | **2,778** | **5,270** | **4,602** | **3,045** | **98,971** | **8,647** | **9,263** | **165** |
| RIPR_008 | 485-508 | Ac-KNSHLHSNIPSLYNSSILPDINKS-amide** | **400** | **2,897** | **48,521** | **65,681** | **9,426** | **92,164** | **216,481** | **700** |
| RIPR_009 | 507-522 | Ac-KSKFHLISRNSRTNQY-amide* | **441** | **635** | **59,072** | **210,134** | **63,414** | **75** | **64** | **15,828** |
| RIPR_011 | 561-577 | Ac-KKVEIFRSRFSSKLQCQ-amide* | **1,361** | **4,423** | **21,996** | **13,691** | **65,618** | **9,217** | **9,427** | **7** |
| RIPR_014 | 662-676 | Ac-DNPYISKYGNKLCER-amide* | **92** | **NB** | **179,379** | **NB** | **95,020** | **191,717** | **262,516** | **25** |
| RIPR_015 | 704-718 | Ac-EEGYKNVKGKCVPDNK-amide** | **1,806** | **112,375** | **NB** | **9,284** | **151,747** | **32,006** | **NB** | **NB** |
| RIPR_017 | 802-817 | Ac-KEHYYRSSRGECILND-amide* | **25** | **27,763** | **NB** | **489** | **6,441** | **1,380** | **NB** | **58,155** |
| RIPR_019 | 1045-1065 | Ac-NEIFHTIIYLKKKIGNSVIYD-amide | **346** | **10,883** | **3,026** | **3,858** | **1,353** | **66** | **3,489** | **898** |

*Peptide trimmed from original ClustiMer output. **Charge added to facilitate peptide synthesis and purification.

Supplementary Table 16. CyRPA Epitope In Vitro Binding Affinity (as IC_50_ in nM) for Class II HLA Alleles.

A subset of predicted CyRPA class II epitope clusters were synthesized as peptides and assessed for HLA-DR binding in vitro utilizing a binding inhibition assay. Note that peptide name relates to the consecutively numbered cluster which the peptide represents in CyRPA.

| **Peptide Name** | **Peptide Address** | **Peptide Sequence** | **DRB1*0101** | **DRB1*0303** | **DRB1*0401** | **DRB1*0701** | **DRB1*0801** | **DRB1*1101** | **DRB1*1301** | **DRB1*1501** |
| --- | --- | --- | --- | --- | --- | --- | --- | --- | --- | --- |
|  |  |  | **IC_50_** | **IC_50_** | **IC_50_** | **IC_50_** | **IC_50_** | **IC_50_** | **IC_50_** | **IC_50_** |
| CYRPA_005 | 240-257 | HFYVGDNINNVKNVNFIE-amide* | **182** | **128** | **78** | **137** | **11,844** | **293** | **633** | **122** |
| CYRPA_008 | 336-355 | Ac-DENRTLFIYSSSQGIYNIHT-amide* | **4** | **1,331** | **1,052** | **219** | **11,515** | **1,127** | **6,984** | **50** |

*Peptide trimmed from original ClustiMer output.

Supplementary Table 17. RH5 Epitope In Vitro Binding Affinity (as IC_50_ in nM) for Class II HLA Alleles.

A subset of predicted RH5 class II epitope clusters were synthesized as peptides and assessed for HLA-DR binding in vitro utilizing a binding inhibition assay. Note that peptide name relates to the consecutively numbered cluster which the peptide represents in RH5. The A and B designations are used when two peptides include sequences from the same cluster.

| **Peptide Name** | **Peptide Address** | **Peptide Sequence** | **DRB1*0101** | **DRB1*0303** | **DRB1*0401** | **DRB1*0701** | **DRB1*0801** | **DRB1*1101** | **DRB1*1301** | **DRB1*1501** |
| --- | --- | --- | --- | --- | --- | --- | --- | --- | --- | --- |
|  |  |  | **IC_50_** | **IC_50_** | **IC_50_** | **IC_50_** | **IC_50_** | **IC_50_** | **IC_50_** | **IC_50_** |
| RH5_003 | 79-97 | Ac-HSTYIKSYLNTNVNDGLKY-amide | **282** | **71,871** | **7,445** | **58,527** | **162,101** | **96,465** | **20,088** | **4** |
| RH5_004 | 93-110 | Ac-DGLKYLFIPSHNSFIKKY-amide* | **4** | **52,171** | **1,611** | **2,202** | **NB** | **3,191** | **NB** | **1,266** |
| RH5_005 | 145-162 | LQYHFKELSNYNIANSID-amide** | **18** | **12,437** | **2,862** | **3,794** | **19,424** | **11,277** | **50,879** | **25** |
| RH5_006A | 179-196 | Ac-YTFLDYYKHLSYNSIYHK-amide* | **122** | **8,477** | **4,394** | **30,454** | **46,845** | **2,449** | **4,353** | **4,131** |
| RH5_006B | 187-205 | Ac-HLSYNSIYHKSSTYGKCIA-amide* | **1,348** | **13,798** | **2,908** | **19765** | **18927** | **834** | **29,280** | **7,804** |
| RH5_008 | 305-326 | Ac-DEYNTKKKKLIKCIKNHENDFN-amide* | **1,001** | **562,770** | **142,707** | **129,239** | **2,595** | **101,759** | **108,201** | **5,144** |
| RH5_009 | 360-381 | Ac-YDEYIHKLILSVKSKNLNKDLS-amide* | **43** | **6,772** | **131,275** | **381** | **6,460** | **10,504** | **88,808** | **10** |
| RH5_010A | 382-397 | DMTNILQQSELLLTNL-amide | **16** | **982** | **1,719** | **1,608** | **NB** | **1,020** | **745** | **789** |
| RH5_010B | 390-407 | SELLLTNLNKKMGSYIYI-amide* | **77** | **100831** | **84,010** | **41,496** | **6,458** | **204** | **9,338** | **9,972** |
| RH5_011 | 453-473 | Ac-DELLKRILDMSNEYSLFITSD-amide | **73** | **10,889** | **2,320** | **13,676** | **95,228** | **30,333** | **37,174** | **2,147** |
| RH5_012 | 488-504 | Ac-KHLNNIFHHLIYVLQMK-amide* | **25** | **7,518** | **7,227** | **24,232** | **91,663** | **33,912** | **8,825** | **7,712** |
| RH5_013 | 512-525 | Ac-EYFQTYKKNKPLTQ-amide | **13,546** | **598,451** | **127,673** | **NB** | **17,834** | **1,112** | **101,465** | **121,270** |

*Peptide trimmed from original ClustiMer output. **Charge added to facilitate peptide synthesis and purification.

Supplementary Table 18. In Vitro Class II HLA Binding Assay: Reference Peptide Sequences and Binding Affinity Color Key.

| **Allele** | **Reference Peptide Sequence** |  | **Class II HLA Allele IC_50_ Binding Affinity Color Key** |
| --- | --- | --- | --- |
| **DRB1*0101** | **PRYVKQNTLKLAT** |  | **Very High Affinity (IC_50_ < 100nM)** |
| **DRB1*0303** | **LFRKDIAAKYKE** |  | **High Affinity (100 nM < IC_50_ < 1,000 nM)** |
| **DRB1*0401** | **YARFQSQTTLKQKT** |  | **Moderate Affinity (1,000 nM < IC_50_ < 10,000 nM)** |
| **DRB1*0701** | **QYIKANSKFIGITEL** |  | **Low Affinity (10,000 nM < IC_50_ < 100,000 nM)** |
| **DRB1*0801** | **KLYRKLKREITFH** |  | **Negligible Affinity (100,000 nM < IC_50_)** |
| **DRB1*1101** | **QYIKANSKFIGITEL** |  | **Non-binder (No dose-dependent inhibition)** |
| **DRB1*1301** | **IELGKKFNIKTRLSYFSMNH** |  |  |
| **DRB1*1501** | **NPVVHFFKNIVTPRTPPPS** |  |  |
| **Negative CTRL** | **ISQAVHAAHAEINEAGR** |  |  |

Supplementary Table 19. Class II Epitope In Silico Predictions Demonstrated an Accuracy of 71% in Predicting Epitopes that Bind HLA-DR Alleles In Vitro.

The Z-score cut-off for the EpiMatrix algorithm is configured to include some peptides with lower binding affinities (increased sensitivity) in order to reduce the possibility of missing high-quality targets (type 2 error). This strategy results in a relatively high false positive rate (reduced specificity).

|  |  |  | **In Silico HLA-DR Binding Prediction  (EpiMatrix Scores ≥ 1.64)** | |
| --- | --- | --- | --- | --- |
|  |  |  | **Positive** | **Negative** |
| **Overall** | **In Vitro HLA-DR Allele Binding  (IC_50_ < 100,000 nM)** | **Positive** | **247  (True Positive)** | **65  (False Negative)** |
|  |  | **Negative** | **40  (False Positive)** | **16  (True Negative)** |
|  |  |  | **Accuracy = 71%** | |
|  |  |  |  | |
|  |  |  | **Positive** | **Negative** |
| **EBA175** | **In Vitro HLA-DR Allele Binding  (IC_50_ < 100,000 nM)** | **Positive** | **62  (True Positive)** | **29  (False Negative)** |
|  |  | **Negative** | **14  (False Positive)** | **7  (True Negative)** |
|  |  |  | **Accuracy = 62%** | |
|  |  |  |  | |
|  |  |  | **Positive** | **Negative** |
| **AMA1** | **In Vitro HLA-DR Allele Binding  (IC_50_ < 100,000 nM)** | **Positive** | **41  (True Positive)** | **12  (False Negative)** |
|  |  | **Negative** | **8  (False Positive)** | **3  (True Negative)** |
|  |  |  | **Accuracy = 69%** | |
|  |  |  |  | |
|  |  |  | **Positive** | **Negative** |
| **RIPR** | **In Vitro HLA-DR Allele Binding  (IC_50_ < 100,000 nM)** | **Positive** | **58  (True Positive)** | **9  (False Negative)** |
|  |  | **Negative** | **8  (False Positive)** | **5  (True Negative)** |
|  |  |  | **Accuracy = 79%** | |
|  |  |  |  | |
|  |  |  | **Positive** | **Negative** |
| **CyRPA** | **In Vitro HLA-DR Allele Binding  (IC_50_ < 100,000 nM)** | **Positive** | **11  (True Positive)** | **0  (False Negative)** |
|  |  | **Negative** | **5  (False Positive)** | **0  (True Negative)** |
|  |  |  | **Accuracy = 69%** | |
|  |  |  |  | |
|  |  |  | **Positive** | **Negative** |
| **RH5** | **In Vitro HLA-DR Allele Binding  (IC_50_ < 100,000 nM)** | **Positive** | **75  (True Positive)** | **15  (False Negative)** |
|  |  | **Negative** | **5  (False Positive)** | **1  (True Negative)** |
|  |  |  | **Accuracy = 79%** | |

Supplementary Table 20. VAC063 Clinical Study Design – Dose Escalation.

Clinical trial participants were healthy malaria-naïve adults residing in the United Kingdom (UK). PBMCs collected from HLA-matched VAC063 donor on study day 42 (14 days post the second vaccine administration) were used for ex vivo IFN-γ recall responses when assessing class II epitope clusters and individual class I epitopes.

| **Phase I** | **Group ID** | **Group Size** | **Day 0** | **Day 28** | **Day 56** | **Day 182** | **# of Donors** | |
| --- | --- | --- | --- | --- | --- | --- | --- | --- |
|  |  |  |  |  |  |  | **Class II** | **Class I** |
| Dose Escalation | 1 | 12 | 2 μg RH5.1 | 2 μg RH5.1 | 2 μg RH5.1 | - | 8 | 2 |
|  | 2 | 12 | 10 μg RH5.1 | 10 μg RH5.1 | 10 μg RH5.1 | - | 5 | 2 |
|  | 3 | 12 | 50 μg RH5.1 | 50 μg RH5.1 | - | 10 μg RH5.1 | 9 | 0 |
|  | 4 | 12 | 50 μg RH5.1 | 50 μg RH5.1 | 50 μg RH5.1 | - | 6 | 1 |
| Group 3 received a delayed fractional third dose of vaccine on day 182. All doses of RH5.1 were administered with 0.5mL of AS01B. | | | | | | | | |

Supplementary Table 21. VAC057 Clinical Study Design – Dose Escalation.

Clinical trial participants were healthy malaria-naïve adults residing in the UK. PBMCs collected from HLA-matched VAC057 donors on study day 14 (14 days post the first vaccine administration) were used for ex vivo IFN-γ recall responses when assessing class II epitope clusters and individual class I epitopes.

| **Phase I** | **Group ID** | **Group Size** | **Day 0** | **Day 56** | **# of Donors** | |
| --- | --- | --- | --- | --- | --- | --- |
|  |  |  |  |  | **Class II** | **Class I** |
| Dose Escalation | 2A | 4 | ChAd63 RH5, 5x10^10^ vp | - | 2 | 0 |
|  | 2B | 8 | ChAd63 RH5, 5x10^10^ vp | MVA RH5, 1x10^8^ pfu | 7 | 3 |
|  | 2C | 8 | ChAd63 RH5, 5x10^10^ vp | MVA RH5, 2x10^8^ pfu | 5 | 3 |
| ChAd63 – simian adenovirus 63 viral vector vaccine platform, MVA – modified vaccinia virus Ankara viral vector vaccine platform, vp – viral particles, pfu – plaque forming units | | | | | | |


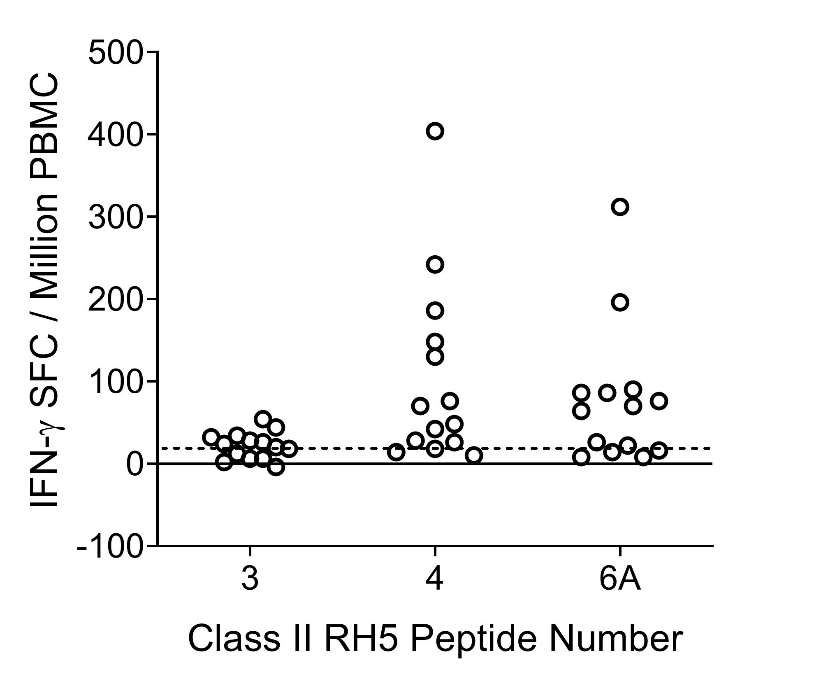


Supplementary Figure 1. VAC057 RH5 Ex Vivo Class II Epitope Cluster IFN-γ Recall Assay – Magnitude of Responses.

The established positivity cut-off (dotted line) was ≥ 20 SFC/million PMBCs.

# Class I Epitope Prediction and Analysis

Supplementary Materials and Methods

Class I Peptide In Vitro Class I HLA Binding Assay

A cell-free class I HLA binding assay that allows for in vitro quantification of peptide-HLA binding affinity in a competition format was used. In this assay, a fluorescently labeled, high binding, control peptide is loaded onto 384-well ½-area plates along with unlabeled experimental peptides. To remove endogenous peptides and render the HLA A and B molecules receptive to binding, HLA is heated at 53°C for 15 minutes and immediately added to the assay. The mixture is then allowed to stand for three days. Once the mixture has reached steady equilibrium (at 72 hours), displacement of the high binding control peptide is measured through fluorescence polarization. Binding of experimental peptides is expressed as the percent inhibition of the labeled control peptide (experimental fluorescence / control fluorescence, multiplied by 100). The percent inhibition values for each experimental peptide (across a range of molar concentrations) are used to calculate the concentration which inhibits 50% of the labeled control peptide’s specific binding. This value is referred to as the peptide’s IC_50_.

Class I Peptide Ex vivo T Cell IFN-γ Recall Assessments

Ex vivo assessments were performed as indicated in the materials and methods section with the exception that class I peptides were used at 10µg/mL.

Supplementary Table 22. In Vitro Class I HLA Binding Assay: Reference Peptide Sequences and Binding Affinity Color Key.

| **Allele** | **Reference Peptide Sequence** | **Negative Control Peptide Sequence** |  | **Class I HLA Allele IC_50_ Binding Affinity Color Key** |
| --- | --- | --- | --- | --- |
| **A*0101** | **IADMGHLKY** | **IPSYKKLIM** |  | **Very High Affinity (IC_50_ < 5,000nM)** |
| **A*0201** | **GLMTTVHAI** | **IPSYKKLIM** |  | **High Affinity (5,000 nM < IC_50_ < 50,000 nM)** |
| **A*0301** | **SLFRAVITK** | **IPSYKKLIM** |  | **Moderate Affinity (50,000 nM < IC_50_ < 350,000 nM)** |
| **A*2402** | **PYVSRLLGI** | **IPSYKKLIM** |  | **Low Affinity (350,000 nM < IC_50_ < 1,000,000 nM)** |
| **B*0702** | **IPSYKKLIM** | **SLFRAVITK** |  | **Negligible Affinity (1,000,000 nM < IC_50_)** |
| **B*4402** | **MEVDPIGHLY** | **SLFRAVITK** |  | **Non-binder (No dose-dependent inhibition)** |

Supplementary Table 23. EBA175 Class I HLA Allele In Vitro Binding Assay Data.

For each predicted class I HLA allele epitope, the EpiMatrix Z-score and in vitro HLA binding affinity (as IC_50_) are shown. Peptides are numbered to indicate the starting amino acid reflective of the protein sequence. For each allele, the rows are sorted by lowest IC_50_ (highest affinity). Refer to **Table S22** for the IC_50_ Binding Affinity Key.

| **Allele** | **Peptide Name** | **Peptide ID** | **EpiMatrix Z-score** | **IC_50_ (nM)** |
| --- | --- | --- | --- | --- |
| **A*0101** | **EBA175_1392** | **FTYDSEEYY** | **3.64** | **136.2** |
| **A*0101** | **EBA175_1084** | **TTERISNNEY** | **4.43** | **904.5** |
| **A*0101** | **EBA175_0114** | **TSSLIKQNKY** | **3.94** | **37092** |
| **A*0101** | **EBA175_1465** | **VTDNLDKLSN** | **3.17** | **187609** |
| **A*0101** | **EBA175_0881** | **LSDTLQLHED** | **3.42** | **Non-binder** |
| **A*0201** | **EBA175_1461** | **FLFEVTDNL** | **3.13** | **269.4** |
| **A*0201** | **EBA175_0697** | **KMYSEFKSI** | **2.57** | **1025** |
| **A*0201** | **EBA175_0896** | **KLPLESSTI** | **1.99** | **2119** |
| **A*0201** | **EBA175_0437** | **VLNGNDNTI** | **2.17** | **7306** |
| **A*0201** | **EBA175_0116** | **SLIKQNKYV** | **2.51** | **29876** |
| **A*0201** | **EBA175_0436** | **SVLNGNDNTI** | **2.16** | **640079** |
| **A*0301** | **EBA175_0069** | **ILNNSKFNK** | **3.33** | **419.9** |
| **A*0301** | **EBA175_0058** | **KIFTFIENK** | **3.12** | **466.7** |
| **A*0301** | **EBA175_1214** | **RLSHTDIHK** | **3** | **1735** |
| **A*0301** | **EBA175_0667** | **NSYKEWISKK** | **3.15** | **7373** |
| **A*2402** | **EBA175_1316** | **RYNLYDKKL** | **2.67** | **171.7** |
| **A*2402** | **EBA175_0896** | **KLPLESSTI** | **2** | **214.1** |
| **A*2402** | **EBA175_0255** | **GYSTKAENKI** | **2.58** | **1838** |
| **A*2402** | **EBA175_1390** | **SYFTYDSEEY** | **2.78** | **22344** |
| **B*0702** | **EBA175_1224** | **NPEDRNSNTL** | **2.37** | **2704** |
| **B*0702** | **EBA175_0979** | **SPNDNINVT** | **2.16** | **36082** |
| **B*0702** | **EBA175_0971** | **MPKAVIGSSP** | **3.01** | **293146** |
| **B*0702** | **EBA175_0794** | **NPQQSVGENG** | **2.55** | **Non-binder** |
| **B*0702** | **EBA175_1073** | **TPGPLDNTSE** | **2.26** | **Non-binder** |
| **B*0702** | **EBA175_1260** | **LQKHGFHTM** | **1.96** | **Non-binder** |
| **B*4402** | **EBA175_0375** | **FEWHTLSKEY** | **3.09** | **19115** |
| **B*4402** | **EBA175_1211** | **EEERLSHTDI** | **3.02** | **19115** |
| **B*4402** | **EBA175_0316** | **EELQITQWI** | **3.79** | **19632** |
| **B*4402** | **EBA175_1454** | **NENNENNFL** | **3.19** | **Non-binder** |

Supplementary Table 24. AMA1 Class I HLA Allele In Vitro Binding Assay Data.

For each predicted class I HLA allele epitope, the EpiMatrix Z-score and in vitro HLA binding affinity (as IC_50_) are shown. Peptides are numbered to indicate the starting amino acid reflective of the protein sequence. For each allele, the rows are sorted by lowest IC_50_ (highest affinity). Refer to **Table S22** for the IC_50_ Binding Affinity Key.

| **Allele** | **Peptide Name** | **Peptide ID** | **EpiMatrix Z-score** | **IC_50_ (nM)** |
| --- | --- | --- | --- | --- |
| **A*0101** | **AMA1_166** | **LTPVATGNQY** | **3.29** | **2559** |
| **A*0101** | **AMA1_044** | **HREHPKEYEY** | **3.38** | **11052** |
| **A*0101** | **AMA1_096** | **SIEIVERSNY** | **3.27** | **12129** |
| **A*0101** | **AMA1_225** | **IPDNDKNSNY** | **3.32** | **14712** |
| **A*0101** | **AMA1_346** | **ASDQPKQYEQ** | **3.27** | **125815** |
| **A*0201** | **AMA1_379** | **FLPTGAFKA** | **2.45** | **1391** |
| **A*0201** | **AMA1_092** | **NLFSSIEIV** | **2.37** | **1555** |
| **A*0201** | **AMA1_418** | **CLINNSSYI** | **2.59** | **3818** |
| **A*0201** | **AMA1_373** | **SMIKSAFLPT** | **1.83** | **12520** |
| **A*0201** | **AMA1_105** | **YMGNPWTEYM** | **2.35** | **21393** |
| **A*0201** | **AMA1_556** | **AVLATILMV** | **2.32** | **23635** |
| **A*0201** | **AMA1_480** | **FISDDKDSL** | **2.06** | **335616** |
| **A*0201** | **AMA1_007** | **VLLLSAFEFT** | **2.26** | **Non-binder** |
| **A*0201** | **AMA1_607** | **KRASHTTPV** | **1.83** | **Non-binder** |
| **A*0301** | **AMA1_377** | **SAFLPTGAFK** | **3.11** | **344.7** |
| **A*0301** | **AMA1_500** | **STCRFFVCK** | **3.37** | **5902** |
| **A*0301** | **AMA1_437** | **VENNFPCSLY** | **2.29** | **85401** |
| **A*0301** | **AMA1_168** | **PVATGNQYLK** | **2.35** | **328427** |
| **A*0301** | **AMA1_480K** | **FISDDKDSLK** | **2.86** | **696624** |
| **A*2402** | **AMA1_174** | **QYLKDGGFAF** | **3.48** | **41.86** |
| **A*2402** | **AMA1_020** | **NFGRGQNYW** | **2.68** | **228.5** |
| **A*2402** | **AMA1_052** | **EYPLHQEHTY** | **3.26** | **998.8** |
| **A*2402** | **AMA1_527** | **EYKDEYADI** | **2.79** | **2102** |
| **A*2402** | **AMA1_104** | **NYMGNPWTE** | **3.4** | **37916** |
| **A*2402** | **AMA1_321** | **EDIPHVNEF** | **2.02** | **83026** |
| **A*2402** | **AMA1_396** | **GYNWGNYNTE** | **2.07** | **Non-binder** |
| **B*0702** | **AMA1_608** | **RASHTTPVL** | **2.52** | **258** |
| **B*0702** | **AMA1_302** | **CPRKNLQNA** | **2.61** | **484.4** |
| **B*0702** | **AMA1_433** | **HPIEVENNF** | **2.43** | **310190** |
| **B*0702** | **AMA1_376** | **KSAFLPTGAF** | **2.07** | **Non-binder** |
| **B*0702** | **AMA1_441** | **FPCSLYKDEI** | **3.1** | **Non-binder** |
| **B*0702** | **AMA1_476** | **APRIFISDD** | **2.01** | **Non-binder** |
| **B*0702** | **AMA1_607** | **KRASHTTPV** | **2.04** | **Non-binder** |
| **B*4402** | **AMA1_437** | **VENNFPCSLY** | **3.66** | **6021** |
| **B*4402** | **AMA1_097** | **IEIVERSNY** | **2.94** | **8031** |
| **B*4402** | **AMA1_604** | **GEEKRASHTT** | **2.33** | **10020** |
| **B*4402** | **AMA1_593** | **DEMLDPEASF** | **2.74** | **11470** |

Supplementary Table 25. RIPR Class I HLA Allele In Vitro Binding Assay Data.

For each predicted class I HLA allele epitope, the EpiMatrix Z-score and in vitro HLA binding affinity (as IC_50_) are shown. Peptides are numbered to indicate the starting amino acid reflective of the protein sequence. For each allele, the rows are sorted by lowest IC_50_ (highest affinity). Refer to **Table S22** for the IC_50_ Binding Affinity Key.

| **Allele** | **Peptide Name** | **Peptide ID** | **EpiMatrix Z-score** | **IC_50_ (nM)** |
| --- | --- | --- | --- | --- |
| **A*0101** | **RIPR_0059** | **ENDYAYLNKY** | **3.29** | **42157** |
| **A*0101** | **RIPR_0648** | **STCYGNRFNY** | **2.91** | **53957** |
| **A*0101** | **RIPR_0545** | **STDPHTNSNN** | **3.28** | **Non-binder** |
| **A*0101** | **RIPR_0611** | **SECDIGLIY** | **2.35** | **Non-binder** |
| **A*0101** | **RIPR_0976** | **DSCVPNTNEY** | **3.25** | **Non-binder** |
| **A*0201** | **RIPR_0991** | **FQYNDDASI** | **2.48** | **275.4** |
| **A*0201** | **RIPR_0064** | **YLNKYVYTI** | **3.54** | **342.1** |
| **A*0201** | **RIPR_1011** | **YIYNQIIWKI** | **3.11** | **1950** |
| **A*0201** | **RIPR_0745** | **FVLENGVCI** | **2.2** | **4174** |
| **A*0201** | **RIPR_0035** | **KLTFSLDHRV** | **2.49** | **8876** |
| **A*0301** | **RIPR_0425** | **KLKYVTESLK** | **3.68** | **3339** |
| **A*0301** | **RIPR_0266** | **ATCKYGFNK** | **3.6** | **4752** |
| **A*0301** | **RIPR_0500** | **SILPDINKSK** | **3.09** | **12710** |
| **A*0301** | **RIPR_0924** | **CVCTYNYYKK** | **3.33** | **14410** |
| **A*0301** | **RIPR_0648** | **STCYGNRFNY** | **3.34** | **Non-binder** |
| **A*2402** | **RIPR_0412** | **AYVSCYRVSF** | **3.42** | **56.89** |
| **A*2402** | **RIPR_0063** | **AYLNKYVYTI** | **3.6** | **127.2** |
| **A*2402** | **RIPR_0064** | **YLNKYVYTI** | **2.42** | **135** |
| **A*2402** | **RIPR_0805** | **YYRSSRGECI** | **3.42** | **175.4** |
| **A*2402** | **RIPR_0664** | **PYISKYGNKL** | **2.53** | **179.5** |
| **B*0702** | **RIPR_0676** | **RPNDCESVL** | **3.39** | **377.3** |
| **B*0702** | **RIPR_0502** | **LPDINKSKF** | **2.21** | **595.2** |
| **B*0702** | **RIPR_0246** | **SPLNLQENEM** | **2.98** | **22388** |
| **B*0702** | **RIPR_0836** | **KPECVCKENL** | **3.19** | **205885** |
| **B*0702** | **RIPR_0745** | **FVLENGVCI** | **2** | **284050** |
| **B*0702** | **RIPR_0313** | **LPSAKNNCEY** | **2.83** | **Non-binder** |
| **B*0702** | **RIPR_0331** | **CPENSTCEQI** | **2.7** | **Non-binder** |
| **B*0702** | **RIPR_0878** | **KPHECVCNKQ** | **2.36** | **Non-binder** |
| **B*4402** | **RIPR_0226** | **YENKSLTHYL** | **3.74** | **112491** |
| **B*4402** | **RIPR_0031** | **NEIDKLTFSL** | **3.98** | **331646** |
| **B*4402** | **RIPR_0283** | **EENYRCTQDI** | **3.64** | **674181** |
| **B*4402** | **RIPR_0378** | **KEKFYKNNLY** | **2.85** | **918865** |
| **B*4402** | **RIPR_0611** | **SECDIGLIY** | **3.08** | **Non-binder** |

Supplementary Table 26. CyRPA Class I HLA Allele In Vitro Binding Assay Data.

For each predicted class I HLA allele epitope, the EpiMatrix Z-score and in vitro HLA binding affinity (as IC_50_) are shown. Peptides are numbered to indicate the starting amino acid reflective of the protein sequence. For each allele, the rows are sorted by lowest IC_50_ (highest affinity). Refer to **Table S22** for the IC_50_ Binding Affinity Key.

| **Allele** | **Peptide Name** | **Peptide ID** | **EpiMatrix Z-score** | **IC_50_ (nM)** |
| --- | --- | --- | --- | --- |
| **A*0101** | **CYRPA_286** | **LNDEYIVSY** | **2.89** | **28126** |
| **A*0101** | **CYRPA_282** | **DVSTLNDEY** | **2.97** | **52183** |
| **A*0101** | **CYRPA_311** | **NSILIKPEKY** | **2.98** | **108692** |
| **A*0101** | **CYRPA_285** | **TLNDEYIVSY** | **2.52** | **212677** |
| **A*0101** | **CYRPA_070** | **GEEDETHIY** | **2.98** | **261203** |
| **A*0201** | **CYRPA_220** | **KLGVQYFFL** | **2.35** | **805.2** |
| **A*0201** | **CYRPA_242** | **YVGDNINNV** | **2.44** | **1818** |
| **A*0201** | **CYRPA_304** | **YIFFNNENSI** | **2.34** | **2825** |
| **A*0201** | **CYRPA_082** | **KVKDSWITL** | **2.02** | **4009** |
| **A*0201** | **CYRPA_223** | **VQYFFLRPYI** | **2.43** | **Non-binder** |
| **A*0301** | **CYRPA_178** | **LICGVSPYK** | **2.91** | **418.7** |
| **A*0301** | **CYRPA_161** | **LSSYVSLPLK** | **3.12** | **654.4** |
| **A*0301** | **CYRPA_076** | **HIYVQKKVK** | **2.15** | **897.3** |
| **A*0301** | **CYRPA_285** | **TLNDEYIVSY** | **2.47** | **304001** |
| **A*0301** | **CYRPA_266** | **FVCSNRDFLK** | **3.2** | **Non-binder** |
| **A*2402** | **CYRPA_004** | **PFHKKFISFF** | **3.13** | **35.64** |
| **A*2402** | **CYRPA_305** | **IFFNNENSI** | **2.6** | **56.89** |
| **A*2402** | **CYRPA_055** | **IYKRELYNI** | **3.67** | **86.77** |
| **A*2402** | **CYRPA_001** | **MIIPFHKKF** | **2.61** | **294.7** |
| **A*2402** | **CYRPA_218** | **NYKLGVQYFF** | **4.44** | **26292** |
| **B*0702** | **CYRPA_229** | **RPYISKNDL** | **3.27** | **243.6** |
| **B*0702** | **CYRPA_082** | **KVKDSWITL** | **2.45** | **458** |
| **B*0702** | **CYRPA_003** | **IPFHKKFISF** | **2.71** | **21878** |
| **B*0702** | **CYRPA_316** | **KPEKYGNTTA** | **2.39** | **835870** |
| **B*0702** | **CYRPA_167** | **LPLKIENREY** | **1.95** | **Non-binder** |
| **B*4402** | **CYRPA_147** | **SEITISDYI** | **3.43** | **5377** |
| **B*4402** | **CYRPA_264** | **LEFVCSNRDF** | **2.73** | **7463** |
| **B*4402** | **CYRPA_142** | **KEYNNSEITI** | **2.99** | **10961** |
| **B*4402** | **CYRPA_223** | **VQYFFLRPYI** | **2.43** | **Non-binder** |
| **B*4402** | **CYRPA_296** | **NDNNFAECY** | **2.92** | **Non-binder** |

Supplementary Table 27. RH5 Class I HLA Allele In Vitro Binding Assay Data.

For each predicted class I HLA allele epitope, the EpiMatrix Z-score and in vitro HLA binding affinity (as IC_50_) are shown. Peptides are numbered to indicate the starting amino acid reflective of the protein sequence. For each allele, the rows are sorted by lowest IC_50_ (highest affinity). Refer to **Table S22** for the IC_50_ Binding Affinity Key.

| **Allele** | **Peptide Name** | **Peptide ID** | **EpiMatrix Z-score** | **IC_50_ (nM)** |
| --- | --- | --- | --- | --- |
| **A*0101** | **RH5_505** | **FNDVPIKMEY** | **3.73** | **498** |
| **A*0101** | **RH5_181** | **FLDYYKHLSY** | **5.08** | **646** |
| **A*0101** | **RH5_406** | **YIDTIKFIHK** | **3.23** | **1507** |
| **A*0101** | **RH5_88** | **NTNVNDGLKY** | **3.22** | **4125** |
| **A*0101** | **RH5_352** | **NTNGIRYHY** | **4.03** | **4381** |
| **A*0101** | **RH5_280** | **DTDSNHTPSN** | **2.97** | **5883** |
| **A*0101** | **RH5_246** | **NKNDDSYRY** | **2.6** | **6677** |
| **A*0101** | **RH5_46** | **STEEEKDDIK** | **2.86** | **6828** |
| **A*0101** | **RH5_130** | **NNEDYKNVDY** | **2.82** | **7991** |
| **A*0101** | **RH5_131** | **NEDYKNVDY** | **2.81** | **8922** |
| **A*0101** | **RH5_170** | **HLDFVIIPHY** | **3.82** | **118129** |
| **A*0101** | **RH5_010** | **LTIIYIHLF** | **2.53** | **Non-binder** |
| **A*0101** | **RH5_338** | **NLFEQLSCY** | **3.02** | **Non-Binder** |
| **A*0101** | **RH5_191** | **NSIYHKSSTY** | **2.72** | **Non-Binder** |
| **A*0101** | **RH5_298** | **RTFKKMMDEY** | **2.62** | **Non-Binder** |
| **A*0101** | **RH5_192** | **SIYHKSSTY** | **2.6** | **Non-Binder** |
| **A*0201** | **RH5_489** | **HLNNIFHHL** | **2.83** | **225** |
| **A*0201** | **RH5_174** | **VIIPHYTFL** | **2.58** | **595** |
| **A*0201** | **RH5_363** | **YIHKLILSV** | **2.91** | **1153** |
| **A*0201** | **RH5_492** | **NIFHHLIYV** | **2.71** | **2285** |
| **A*0201** | **RH5_86** | **YLNTNVNDGL** | **2.2** | **2834** |
| **A*0201** | **RH5_459** | **ILDMSNEYSL** | **2.35** | **6870** |
| **A*0201** | **RH5_467** | **SLFITSDHL** | **2.32** | **7121** |
| **A*0201** | **RH5_212** | **KINETYDKV** | **2.09** | **10071** |
| **A*0201** | **RH5_144** | **FLQYHFKEL** | **2.51** | **14558** |
| **A*0201** | **RH5_99** | **FIPSHNSFI** | **2.74** | **30952** |
| **A*0201** | **RH5_404** | **YIYIDTIKFI** | **2.48** | **53094** |
| **A*0201** | **RH5_476** | **RQMLYNTFYS** | **1.9** | **319024** |
| **A*0201** | **RH5_302** | **KMMDEYNTKK** | **1.75** | **511314** |
| **A*0201** | **RH5_461** | **DMSNEYSLFI** | **2.29** | **Non-Binder** |
| **A*0301** | **RH5_443** | **KLNIWRTFQK** | **3.63** | **2498** |
| **A*0301** | **RH5_188** | **LSYNSIYHK** | **3.08** | **3067** |
| **A*0301** | **RH5_192** | **SIYHKSSTY** | **2.96** | **5933** |
| **A*0301** | **RH5_302** | **KMMDEYNTKK** | **3.97** | **8477** |
| **A*0301** | **RH5_392** | **LLLTNLNKK** | **2.74** | **14121** |
| **A*0301** | **RH5_448** | **RTFQKDELLK** | **2.63** | **24189** |
| **A*0301** | **RH5_219** | **KVKSKCNDIK** | **3.2** | **39323** |
| **A*0301** | **RH5_187** | **HLSYNSIYHK** | **3.55** | **44197** |
| **A*0301** | **RH5_402** | **GSYIYIDTIK** | **3.4** | **70759** |
| **A*0301** | **RH5_427** | **HTKIINDKTK** | **2.35** | **72993** |
| **A*0301** | **RH5_191** | **NSIYHKSSTY** | **2.52** | **100781** |
| **A*0301** | **RH5_99** | **FIPSHNSFIK** | **3.06** | **Non-Binder** |
| **A*0301** | **RH5_294** | **DLMNRTFKK** | **3.04** | **Non-Binder** |
| **A*0301** | **RH5_298** | **RTFKKMMDEY** | **2.85** | **Non-Binder** |
| **A*0301** | **RH5_141** | **NVNFLQYHFK** | **2.82** | **Non-Binder** |
| **A*0301** | **RH5_181** | **FLDYYKHLSY** | **2.55** | **Non-Binder** |
| **A*0301** | **RH5_369** | **LSVKSKNLNK** | **2.56** | **Non-Binder** |
| **A*0301** | **RH5_101** | **PSHNSFIKK** | **2.34** | **Non-Binder** |
| **A*2402** | **RH5_105** | **SFIKKYSVF** | **3.16** | **38.3** |
| **A*2402** | **RH5_098** | **LFIPSHNSF** | **3.76** | **141.2** |
| **A*2402** | **RH5_180** | **TFLDYYKHL** | **2.69** | **215.4** |
| **A*2402** | **RH5_172** | **DFVIIPHYTF** | **3.41** | **288.7** |
| **A*2402** | **RH5_251** | **SYRYDISEEI** | **2.81** | **362.8** |
| **A*2402** | **RH5_446** | **IWRTFQKDEL** | **2.88** | **625** |
| **A*2402** | **RH5_442** | **IKLNIWRTF** | **2.45** | **1420** |
| **A*2402** | **RH5_331** | **DMKNYGTNLF** | **2.75** | **5305** |
| **A*2402** | **RH5_096** | **KYLFIPSHN** | **2.4** | **6124** |
| **A*2402** | **RH5_493** | **IFHHLIYVL** | **2.55** | **9587** |
| **A*2402** | **RH5_189** | **SYNSIYHKS** | **2.32** | **57735** |
| **A*2402** | **RH5_010** | **LTIIYIHLF** | **2.53** | **69520** |
| **A*2402** | **RH5_013** | **IYIHLFILN** | **2.41** | **141575** |
| **B*0702** | **RH5_286** | **TPSNKKKNDL** | **2.66** | **1879** |
| **B*0702** | **RH5_173** | **FVIIPHYTF** | **1.87** | **3304** |
| **B*0702** | **RH5_011** | **TIIYIHLFIL** | **1.8** | **13708** |
| **B*0702** | **RH5_004** | **IKKKLILTII** | **1.73** | **44149** |
| **B*0702** | **RH5_470** | **ITSDHLRQM** | **1.74** | **183252** |
| **B*0702** | **RH5_001** | **MIRIKKKLIL** | **1.79** | **Non-binder** |
| **B*0702** | **RH5_042** | **LPIKSTEEEK** | **1.67** | **Non-binder** |
| **B*0702** | **RH5_176** | **IPHYTFLDYY** | **1.98** | **Non-binder** |
| **B*0702** | **RH5_240** | **HPYDINNKND** | **2.13** | **Non-binder** |
| **B*0702** | **RH5_296** | **MNRTFKKMM** | **1.81** | **Non-binder** |
| **B*0702** | **RH5_330** | **MDMKNYGTNL** | **2.42** | **Non-binder** |
| **B*0702** | **RH5_421** | **FNRIEYHTKI** | **1.89** | **Non-binder** |
| **B*0702** | **RH5_469** | **FITSDHLRQM** | **1.64** | **Non-binder** |
| **B*4402** | **RH5_415** | **KEMKHIFNRI** | **2.76** | **39177** |
| **B*4402** | **RH5_049** | **EEKDDIKNG** | **2.62** | **Non-binder** |
| **B*4402** | **RH5_062** | **KEIDNDKENI** | **2.83** | **Non-binder** |
| **B*4402** | **RH5_123** | **NEKNDVKNN** | **1.99** | **Non-binder** |
| **B*4402** | **RH5_361** | **DEYIHKLIL** | **2.33** | **Non-binder** |
| **B*4402** | **RH5_378** | **KDLSDMTNI** | **2.56** | **Non-binder** |
| **B*4402** | **RH5_464** | **NEYSLFITS** | **2.55** | **Non-binder** |
| **B*4402** | **RH5_486** | **KEKHLNNIF** | **2.27** | **Non-binder** |
| **B*4402** | **RH5_506** | **NDVPIKMEYF** | **2.37** | **Non-binder** |

Supplementary Table 28. Class I Epitope In Silico Predictions Demonstrated an Accuracy of 75% in Predicting Epitopes that Bind HLA-A and HLA-B Alleles In Vitro.

Confirmed class I HLA allele binding for predicted epitopes ranged from 51% to 97% across the set of alleles evaluated. Overall, 75% of the predicted epitopes tested bound the class I HLA allele (in vitro) that they were predicted to bind.

|  |  |  | **Binders** | | | |  |  |
| --- | --- | --- | --- | --- | --- | --- | --- | --- |
| **Allele** | **Total Number peptides tested** | **Total Binders** | **Very High Affinity** | **High Affinity** | **Moderate Affinity** | **Low Affinity** | **Total Non- Binders*** | **Confirmed Binders (%)** |
| **A*0101** | **36** | **27** | **8** | **11** | **8** | **0** | **9** | **75** |
| **A*0201** | **39** | **35** | **19** | **11** | **3** | **2** | **4** | **90** |
| **A*0301** | **37** | **28** | **11** | **10** | **6** | **1** | **9** | **76** |
| **A*2402** | **34** | **33** | **23** | **6** | **4** | **0** | **1** | **97** |
| **B*0702** | **39** | **20** | **9** | **5** | **5** | **1** | **19** | **51** |
| **B*4402** | **27** | **15** | **0** | **11** | **2** | **2** | **12** | **56** |
| **TOTAL** | **212** | **158** | **70** | **54** | **28** | **6** | **54** | **75** |
| *Total non-binders include peptides with negligible binding and no binding. | | | | | | | | |

Supplementary Table 29. Positive Donor Responses in the RH5 Class I HLA-A*0201 Ex Vivo IFN-γ T Cell Recall Assay.

A total of eleven HLA-matched donors were assessed for IFN-γ recall responses to the set of 14 RH5 peptides (representing 14 class I epitopes) predicted to bind HLA-A*0201 (**Table S27**). Five donors from VAC063 (groups 2 and 4) and six donors from VAC057 were included. All donors were assessed with all peptides. Data were background subtracted prior to analysis and the ≥ 20 SFC/million PBMCs positivity threshold applied. The number of positive donor responses for each peptide is indicated along with the average response (SFC/million PBMCs). Additionally, where the peptides (class I epitopes) overlap the set of RH5 class II clusters, the class II cluster number has been indicated. Not applicable (N/A) is indicated if the class I epitope does not overlap a class II cluster.

| **Peptide #** | **86** | **99** | **144** | **174** | **212** | **302** | **363** | **404** | **459** | **461** | **467** | **476** | **489** | **492** |
| --- | --- | --- | --- | --- | --- | --- | --- | --- | --- | --- | --- | --- | --- | --- |
| **# Positive Donors** | **0** | **0** | **0** | **0** | **0** | **0** | **2** | **2** | **2** | **1** | **0** | **0** | **0** | **4** |
| **Average Response** | **-2** | **-5** | **-3** | **-4** | **-4** | **-6** | **11** | **19** | **20** | **1** | **-5** | **-3** | **-2** | **175** |
| **Class II Cluster #** | **3** | **4** | **5** | **N/A** | **N/A** | **8** | **10** | **N/A** | **11** | **11** | **11** | **N/A** | **12** | **12** |

Supplementary Figure 2. Magnitude of Donor Responses in the RH5 Class I HLA-A*0201 Ex Vivo IFN-γ T Cell Recall Assay.

A total of eleven HLA-matched donors were assessed for IFN-γ recall responses to the set of 14 RH5 peptides predicted to bind HLA-A*0201 (**Table S27**). Five donors from VAC063 (groups 2 and 4) and six donors from VAC057 were included. All donors were assessed with all peptides. Data were background subtracted prior to analysis and the ≥ 20 SFC/million PBMCs positivity threshold applied (dotted line). Positive responses were seen with five peptides 363, 404, 459, 461, and 492 with the highest magnitude of response seen to peptide 492, to which four of the VAC057 donors responded.
